# Effect of Denosumab or Alendronic Acid on the Progression of Aortic Stenosis: A Double-Blind Randomized Controlled Trial

**DOI:** 10.1161/CIRCULATIONAHA.121.053708

**Published:** 2021-04-29

**Authors:** Tania A. Pawade, Mhairi K. Doris, Rong Bing, Audrey C. White, Laura Forsyth, Emily Evans, Catriona Graham, Michelle C. Williams, Edwin J.R. van Beek, Alison Fletcher, Philip D. Adamson, Jack P.M. Andrews, Timothy R.G. Cartlidge, William S.A. Jenkins, Maaz Syed, Takeshi Fujisawa, Christophe Lucatelli, William Fraser, Stuart H. Ralston, Nicholas Boon, Bernard Prendergast, David E. Newby, Marc R. Dweck

**Affiliations:** 1 British Heart Foundation Centre for Cardiovascular Science (T.A.P., M.K.D., R.B., A.C.W., M.C.W., P.D.A., J.P.M.A., T.R.G.C., W.S.A.J., M.S., T.F., N.B., D.E.N., M.R.D.), University of Edinburgh, United Kingdom.; 2 Edinburgh Clinical Trials Unit (L.F.), University of Edinburgh, United Kingdom.; 3 Edinburgh Clinical Research Facility (E.E., C.G.), University of Edinburgh, United Kingdom.; 4 Edinburgh Imaging (E.J.R.v.B., A.F., C.L.), University of Edinburgh, United Kingdom.; 5 Institute of Genetics and Molecular Medicine (S.H.R.), University of Edinburgh, United Kingdom.; 6 Christchurch Heart Institute, University of Otago, New Zealand (P.D.A.).; 7 Norwich Medical School, University of East Anglia, United Kingdom (W.F.).; 8 King’s College London, United Kingdom (B.P.).

**Keywords:** alendronate, aortic stenosis, computed tomography, X-ray, calcium signaling, denosumab

## Abstract

Valvular calcification is central to the pathogenesis and progression of aortic stenosis, with preclinical and observational studies suggesting that bone turnover and osteoblastic differentiation of valvular interstitial cells are important contributory mechanisms. We aimed to establish whether inhibition of these pathways with denosumab or alendronic acid could reduce disease progression in aortic stenosis.

**Methods:**

In a single-center, parallel group, double-blind randomized controlled trial, patients >50 years of age with calcific aortic stenosis (peak aortic jet velocity >2.5 m/s) were randomized 2:1:2:1 to denosumab (60 mg every 6 months), placebo injection, alendronic acid (70 mg once weekly), or placebo capsule. Participants underwent serial assessments with Doppler echocardiography, computed tomography aortic valve calcium scoring, and ^18^F-sodium fluoride positron emission tomography and computed tomography. The primary end point was the calculated 24-month change in aortic valve calcium score.

**Results:**

A total of 150 patients (mean age, 72±8 years; 21% women) with calcific aortic stenosis (peak aortic jet velocity, 3.36 m/s [2.93–3.82 m/s]; aortic valve calcium score, 1152 AU [655–2065 AU]) were randomized and received the allocated trial intervention: denosumab (n=49), alendronic acid (n=51), and placebo (injection n=25, capsule n=25; pooled for analysis). Serum C-terminal telopeptide, a measure of bone turnover, halved from baseline to 6 months with denosumab (0.23 [0.18–0.33 µg/L] to 0.11 µg/L [0.08–0.17 µg/L]) and alendronic acid (0.20 [0.14–0.28 µg/L] to 0.09 µg/L [0.08–0.13 µg/L]) but was unchanged with placebo (0.23 [0.17–0.30 µg/L] to 0.26 µg/L [0.16–0.31 µg/L]). There were no differences in 24-month change in aortic valve calcium score between denosumab and placebo (343 [198–804 AU] versus 354 AU [76–675 AU]; P=0.41) or alendronic acid and placebo (326 [138–813 AU] versus 354 AU [76–675 AU]; *P*=0.49). Similarly, there were no differences in change in peak aortic jet velocity or ^18^F-sodium fluoride aortic valve uptake.

**Conclusions:**

Neither denosumab nor alendronic acid affected progression of aortic valve calcification in patients with calcific aortic stenosis. Alternative pathways and mechanisms need to be explored to identify disease-modifying therapies for the growing population of patients with this potentially fatal condition.

**Registration:**

URL: https://www.clinicaltrials.gov; Unique identifier: NCT02132026.

## Abstract 2

Supplemental Digital Content is available in the text.

**Clinical Perspective**

**What Is New?**

- Active calcification in the aortic valve has been recognized to be a major determinant of disease progression in aortic stenosis.
- This is the first double-blind, randomized controlled trial to test whether drugs targeting processes of active calcification, denosumab or alendronic acid, could slow the progression of aortic stenosis.
- We found that denosumab and alendronate have no major effect on the progression of aortic stenosis as assessed by echocardiography, computed tomography, or ^18^F-sodium fluoride positron emission tomography.

**What Are the Clinical Implications?**

- Neither denosumab nor alendronate cause major amelioration or acceleration of aortic valve calcification or disease progression.
- Other pathways need to be explored in order identify an effective therapy for this unmet clinical need.

**Editorial, see p 2428**

Despite decades of research and several randomized controlled trials,^1–3^ aortic stenosis remains a disease without an effective medical treatment. Prolonged longevity and the subsequent aging of the general population mean that the prevalence of aortic stenosis continues to rise. This has led to the increased use of valve replacement interventions, which remain the only treatment for end-stage disease, although they carry potentially significant periprocedural and long-term risks.^4,5^ A medical therapy that slows the progression of aortic stenosis would therefore be a major advance that addresses an important unmet clinical need.

The pathology of aortic stenosis is driven by actively regulated inflammation and calcification and has clear similarities to skeletal bone formation.^6–10^ Activity of both osteoblasts in the valve and osteoclasts in the bone appears to be important, with multiple potential pathways involved in regulating bone turnover and valvular calcification.^9,11^The receptor activator of nuclear κB ligand (RANKL)/receptor activator of nuclear κB/osteoprotegerin axis is one such pathway. In the valve, RANKL binding stimulates osteogenic differentiation of valvular interstitial cell into osteoblasts, leading to the formation of calcific nodules and expression of alkaline phosphatase and osteocalcin. In the bone, RANKL binding stimulates osteoclastic activity, causing the release of calcium and phosphate into the cardiovascular system. In murine models, targeted inactivation of osteoprotegerin, a decoy receptor for RANKL, leads to widespread vascular calcification and severe osteoporosis, which can be rescued by administration of osteoprotegerin.^12,13^ Preclinical studies also demonstrate that bisphosphonates can reduce the production of proinflammatory cytokines, decrease bone osteoclastic activity, and inhibit arterial, as well as valvular, calcification.^14^ Mechanistic clinical data have demonstrated a link between aortic stenosis progression, vitamin D, and bone remodeling,^15^ while observational clinical studies have demonstrated an association between bisphosphonate use and reduced aortic stenosis progression and coronary calcification.^16–18^ Further support comes from the Multi-Ethnic Study of Atherosclerosis registry, which found that bisphosphonate use was associated with a lower prevalence of cardiovascular calcification (defined as the prevalence of aortic valve, aortic valve ring, mitral annulus, thoracic aorta, and coronary artery calcification on computed tomography [CT]) in women >65 years of age.^19^ However, such associations are not a universal finding,^20^ and a causal relationship cannot be established without randomized controlled trial data.

We have demonstrated the active nature of aortic valve degeneration with in vivo ^18^F-sodium fluoride (^18^F-NaF) positron emission tomography (PET)-CT.^21^ ^18^F-NaF is a bone tracer that binds to hydroxyapatite, a key crystalline component of valvular calcification, which has a greater surface area in regions of developing microscopic calcification. Higher valvular ^18^F-NaF uptake is independently associated with more rapid disease progression and therefore represents a potential biomarker of aortic stenosis disease activity.^22^

On the basis of these data, we conducted the Study Investigating the Effect of Drugs Used to Treat Osteoporosis on the Progression of Calcific Aortic Stenosis (SALTIRE2) randomized controlled trial to determine whether the RANKL inhibitor denosumab or the bisphosphonate alendronic acid could reduce disease progression in patients with calcific aortic stenosis.

## Methods

### Trial Design and Population

This was a single-center, parallel-group, double-blind, randomized controlled trial. The Trial Steering Committee oversaw the conduct and progress of the trial. All patients provided written informed consent. The study was conducted in accordance with the Declaration of Helsinki, approved by the regional ethics committee (Scotland A Research Ethics Committee, 14/SS/0064), and registered on clinicaltrials.gov (Unique identifier: NCT02132026). The study data are not currently in a public repository but may be made available to researchers on reasonable request.

Patients >50 years of age with a peak aortic jet velocity >2.5 m/s on Doppler echocardiography and grade 2 to 4 aortic valve calcification on semiquantitative echocardiographic assessment^23^ were identified from cardiology outpatient clinics across Scotland: Edinburgh Heart Center, Borders General Hospital, Victoria Hospital, Ninewells Hospital, and Forth Valley Royal Hospital. Exclusion criteria were anticipated or planned aortic valve surgery in the next 6 months; life expectancy <2 years; inability to undergo scanning; treatment for osteoporosis with bisphosphonates or denosumab; long-term corticosteroid use; abnormalities of the esophagus or conditions that delay gastric emptying; inability to sit or stand for ≥30 minutes; known allergy or intolerance to alendronic acid, denosumab, or any of their excipients; hypocalcemia; regular calcium supplementation; dental extraction within 6 months; history of osteonecrosis of the jaw; major or untreated cancers; poor dental hygiene; women of childbearing potential who had experienced menarche, were premenopausal, had not been sterilized, or were pregnant; women who were breastfeeding; chronic kidney disease (estimated glomerular filtration rate of <30 mL/min/1.73 m^2^); allergy or contraindication to iodinated contrast; inability or unwillingness to give informed consent; or a likelihood of noncompliance to treatment allocation or study protocol.

### Trial Protocol

Participants underwent clinical history and examination, 6-minute walk test, blood sampling, 12-lead ECG, echocardiography, combined ^18^F-NaF PET-CT, and noncontrast CT. Participants were randomized using computer-based randomization (Edinburgh Clinical Trials Unit, University of Edinburgh) to ensure allocation concealment 4 to 8 weeks after the baseline visit. Patients were allocated to 1 of 4 groups: subcutaneous denosumab (Prolia, Amgen, CA) 60 mg every 6 months, placebo injection every 6 months, oral alendronic acid (TEVA UK, United Kingdom) 70 mg once weekly, or matching placebo capsule once weekly, in a 2:1:2:1 ratio using a minimization algorithm that incorporated a random component. Minimization criteria were age (<73 and ≥73 years), sex, presence or absence of a bicuspid valve, and baseline aortic valve calcium scores (≤1607 and >1607 AU). Participants were randomized in advance of their randomization visit to ensure that the study drug was available to be dispensed at the visit. The placebo capsule contained lactose monohydrate and was manufactured by the Investigational Supplies Group (University of Edinburgh) to be indistinguishable from the encapsulated alendronic acid used. The placebo injection was 0.9% saline, with drug preparation and administration for the injection undertaken by a nominated group of research nurses who remained unblinded. The injection syringes were masked to ensure that patients and the research team remained blinded to treatment allocation. Compliance was calculated as the proportion of expected treatment received for the duration of study participation. For participants in the capsule arms, 32 capsules were given to the participant at each study visit (6-month intervals), and any unused capsules were returned at the subsequent visit, with the assumption that any unreturned capsules were taken as prescribed.

A telephone visit was undertaken 2 weeks after randomization to assess for symptoms of hypocalcemia. Further study follow-up visits were performed at 6, 12, 18, and 24 months, where clinical examination, ECG, echocardiogram, and blood sampling were undertaken. Serum C-terminal telopeptide, a marker of bone resorption, was measured at baseline and 6 months. Repeat ^18^F-NaF PET-CT and noncontrast CT were performed at 12 months and repeat noncontrast CT performed at 24 months. Where possible, participants who were subsequently scheduled for aortic valve replacement had their pending 12- or 24-month visit brought forward, after which the trial intervention was discontinued and no further trial imaging was performed.

### Trial Procedures

#### Echocardiography

All study echocardiograms were performed by a single dedicated research ultrasonographer (A.C.W.) or cardiology research fellow (T.A.P.) on the same echocardiography machine, in the same accredited department using a standardized protocol according to international guidelines. Standard 2-dimensional views and pulsed and continuous wave Doppler measurements were acquired, with Doppler measurements averaged over 3 cardiac cycles or 5 if the patient was in atrial fibrillation.^24^ Aortic valve mean pressure gradient was calculated using the Bernoulli equation. Aortic valve area was estimated using the continuity equation. Aortic stenosis was categorized using standard definitions for peak velocity (mild: 2.6–2.9, moderate: 3.0–4.0, severe: >4.0 m/s) and mean gradient (mild: <20, moderate: 20–40, severe: >40 mm Hg).^25^ Ejection fraction was visually estimated and categorized as normal (≥55%), mildly impaired (45%–54%), moderately impaired (36%–44%), or severely impaired (≤35%).^26^

#### Noncontrast CT and Combined PET-CT

Unless contraindicated, intravenous or oral metoprolol was administered to patients with a heart rate >65 beats per minute. Imaging was performed on a 128-multislice scanner (Biograph mCT, Siemens, Germany) in a dedicated research imaging center (Edinburgh Imaging Facility, University of Edinburgh). Noncontrast CT was performed at baseline, 12 months, and 24 months using the same scanner, along with ECG gating and a standardized protocol (120 kV CARE Dose4D [Siemens], 3-mm slice thickness, spiral acquisition, 70% R-R interval, inspiratory breath-hold). ^18^F-NaF PET-CT was performed on the same scanner at baseline and 12 months. PET image acquisition was performed ≈60 minutes after intravenous injection of 125 MBq ^18^F-NaF with a single bed position centered on the aortic valve. Intravenous iodinated contrast (80 mL Iomeron-400, Bracco Imaging, Italy) was given after PET acquisition, followed by prospective ECG-gated contrast CT acquisition in diastole (CARE Dose4D, Siemens; 0.75-mm slice thickness, spiral acquisition, 50%–75% R-R interval, expiratory breath-hold). Image analysis was performed using Vitrea v6.9.68.1 (Vitrea Advanced, Vital Images, Minnetonka, MN) and FusionQuant v1.20.05.14 (Cedars-Sinai, CA). Aortic valve calcium scores were measured using a standardized technique,^27^ with regions of interest drawn around areas of valvular calcification on sequential axial slices. Care was taken to exclude calcification in adjacent structures, such as the left ventricular outflow tract or sinuses of Valsalva. A standard threshold of 130 Hounsfield units was used to define calcification. The Agatston score was semiautomatically calculated by the software using standard weightings.^28^ ^18^F-NaF aortic valve uptake was measured using a standardized technique of valve orientation en face after coregistration of PET and contrast CT based on blood pool uptake in the cardiac chambers.^29,30^ The valve region of interest was defined by a polyhedron 6 mm in height, centered on the valvular region of highest visual uptake in the *z* plane and contoured manually around the valve perimeter. Blood pool activity was calculated from a 2 cm^2^ region drawn at the center of the right atrium at the level of the right coronary artery ostium. The mean and maximum target to background ratios were calculated by dividing the mean and maximum standardized uptake values in the region of interest by the mean blood pool standardized uptake value. These measures have excellent reproducibility.^24,29,31^

### Trial End Points

The primary end point was the calculated change in aortic valve calcium score at 24 months. Key secondary end points included change in peak aortic jet velocity at 24 months and change in aortic valve uptake at 12 months. The primary end point was calculated as follows: [(final visit aortic valve calcium score – baseline visit aortic valve calcium score)/days from baseline visit to final visit] × 730. Where the participant did not attend a 24-month visit but did attend a 12-month visit, the 12-month visit was used as the final visit. Other imaging end points were calculated in the same way. In the case of end points with 12-month change (aortic valve ^18^F-NaF uptake), the daily rate of change was multiplied by 365 rather than 730.

### Statistical Analysis

To be clinically meaningful, we posited that a disease-modifying therapy would need to delay the time to surgery by 1 to 2 years. In the previous SEAS trial (Simvastatin and Ezetimibe in Aortic Stenosis),^2^ ≈40% of trial participants either died (11%) or underwent aortic valve replacement (30%) within 4 years, with an overall rate of disease progression of 0.61±0.59 m/s. This suggests that we would need to see a difference in the rates of disease progression of ≈40% (from 0.16 to 0.10 m/s/year) to delay the need for surgery by 1 to 2 years. On the basis of aortic valve calcium score progression in participants from our previous studies^1,22^ who had an aortic valve calcium score ≥400 (median 2-year change, 565 AU [interquartile range, 190–910]), we calculated that a sample size of 47 participants would be required per group to detect a 40% difference in the primary end point, with a 2-sided 5% level of significance and 80% power. To account for missing data, the total study sample size was increased to 150. For the primary end point and secondary imaging end points, 24- or 12-month change was calculated on the basis of a daily rate of change from baseline to the relevant follow-up scan, using an intention-to-treat analysis regardless of compliance. Both placebo groups were combined for analysis. For the primary end point, if the baseline noncontrast CT scan was degraded by artifact but 12- and 24-month scans were available, these scans were used to determine the daily rate of change. Sensitivity analyses for the primary end point were performed after excluding scans with artifact or incorporating only those participants with at least 50% or 70% compliance.

Categorical variables are presented as number (percentage), whereas continuous variables are presented as median (interquartile range) or mean±SD. Distributions of data were tested for normality with the Shapiro–Wilk test and quantile-quantile plots. Between-group differences were compared with the Wilcoxon rank-sum test or Kruskal–Wallis test as appropriate. To take into account repeated measurements, mixed-effects linear regression models were constructed for each treatment arm with aortic valve calcium score as the dependent variable, study arm and time point as fixed effects, and participant as a random effect. Least-square means for each active trial arm model were calculated and compared with placebo separately. Spearman’s rank correlation coefficient was performed to assess the relationship between continuous variables. Analysis was performed using SAS Enterprise Guide version 7.15 (SAS Institute Inc., Cary, NC). A 2-sided *p* value of <0.05 was considered statistically significant.

## Results

### Trial Population

**Figure 1.**
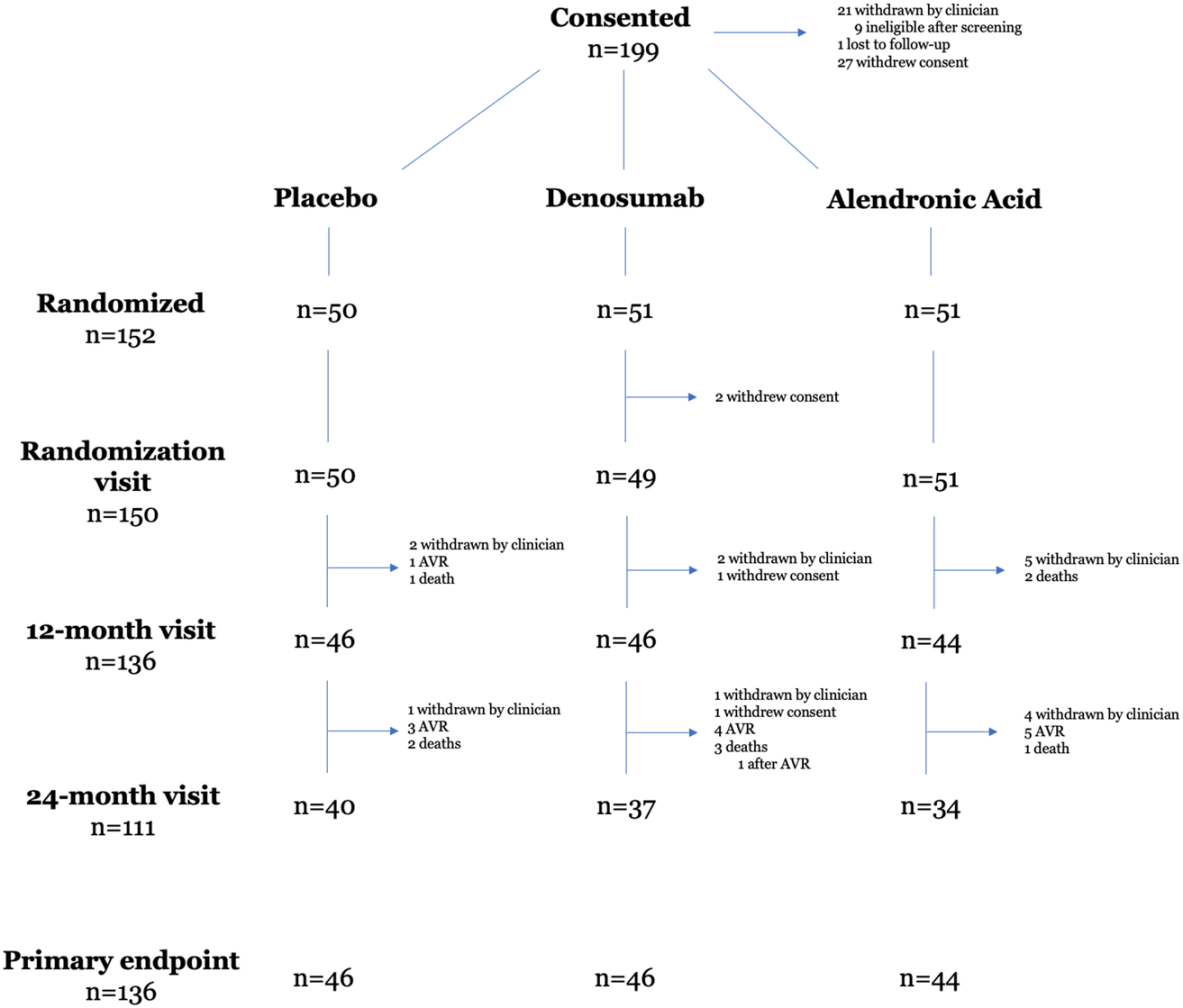
**CONSORT diagram.** The primary end point (24-month change in aortic valve calcium score) was calculated from a daily rate of change based on the difference between baseline and final aortic valve calcium score, whether this was at 12 months or 24 months. AVR indicates aortic valve replacement.

Between August 19, 2015, and November 6, 2017, 199 patients consented, of whom 152 were randomized to denosumab, alendronic acid, or matched placebo. Two participants who were randomized to denosumab and were unaware of their study allocation did not attend the randomization visit or participate further in the study, leaving 150 participants for inclusion in the final analysis (Figure 1).

Baseline characteristics were balanced between study arms (Table). The mean age was 72±8 years, 21% of the cohort were women, and most were of White Scottish ethnicity. There was a high prevalence of hypertension and hypercholesterolemia. The median aortic valve peak velocity and mean gradient were 3.36 m/s (2.93–3.82 m/s) and 23 mm Hg (18–32 mm Hg), respectively. The median aortic valve calcium score was 1152 AU (655–2065 AU; Table).

**Table.**
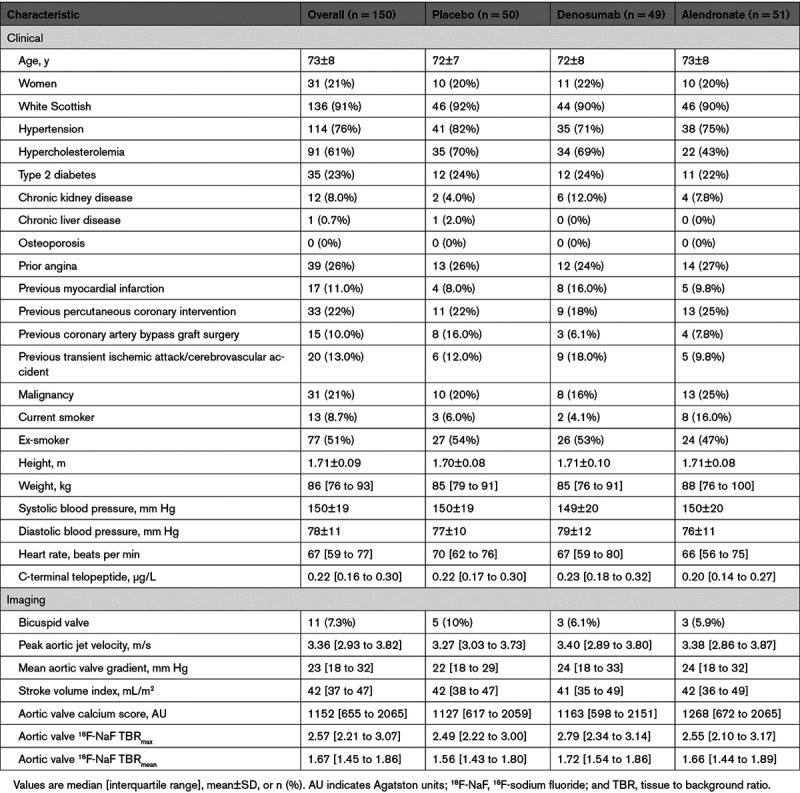
Baseline Characteristics

### Trial Intervention

**Figure 2.**
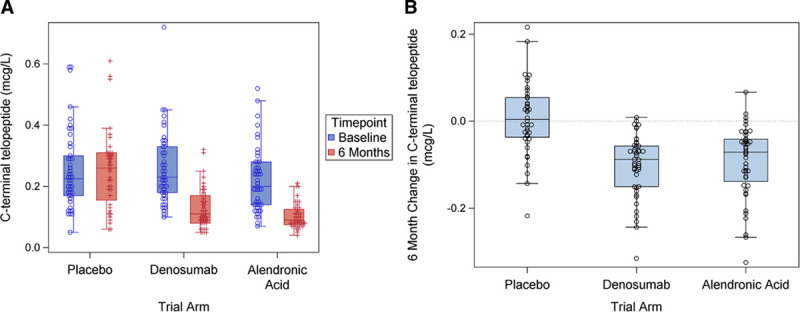
**C-terminal telopeptide concentrations.** **A**, C-terminal telopeptide concentrations at baseline and 6 months for each trial arm (placebo: *P*>0.5, denosumab: *P*<0.001, alendronic acid: *P*<0.001; Wilcoxon rank-sum test). **B**, Six-month change in C-terminal telopeptide for each trial arm (*P*<0.001 for both denosumab and alendronic acid compared to placebo; Wilcoxon rank-sum test).

Compliance was similar between placebo and active drug for each method of administration (proportion of participants receiving >70% expected dose: denosumab 94%, placebo injection 92%, alendronic acid 88%, placebo capsule 84%). Baseline serum C-terminal telopeptide concentrations were similar between treatment arms (Table) and halved from baseline to 6 months with denosumab (0.23 [0.18–0.33 µg/L] to 0.11 µg/L [0.08–0.17 µg/L]) and alendronic acid (0.20 [0.14–0.28 µg/L] to 0.09 µg/L [0.08–0.13 µg/L]) but were unchanged with placebo (0.23 [0.17–0.30 µg/L] to 0.26 µg/L [0.16–0.31 µg/L]; Figure 2).

### Primary End Point

**Figure 3.**
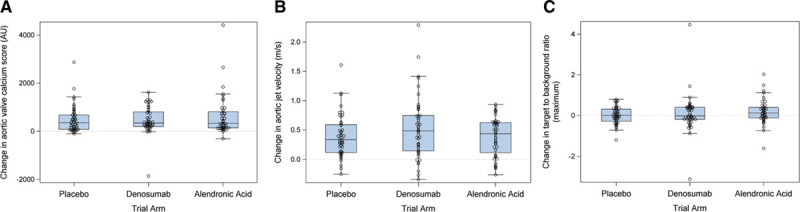
**Primary and key secondary end points.** **A**, Calculated change in 24-month aortic valve calcium score (*P*=0.41 for denosumab versus placebo; *P*=0.49 for alendronic acid versus placebo; Wilcoxon rank-sum test). **B**, Calculated change in 24-month peak aortic jet velocity (*P*=0.21 for denosumab versus placebo; *P*=0.74 for alendronic acid versus placebo; Wilcoxon rank-sum test). **C**, Calculated change in 12-month aortic valve maximum target to background ratio (*P*=0.61 for denosumab versus placebo; *P*=0.15 for alendronic acid versus placebo; Wilcoxon rank-sum test).

The primary end point was calculated in 136 participants (46 placebo, 46 denosumab, 44 alendronic acid; Figure 1) with a median time to final scan of 784 days (770–793 days), in whom the overall aortic valve calcium score at baseline was 1110 AU (622–1998 AU). Compared with placebo, there were no differences in the 24-month change in aortic valve calcium score for either denosumab or alendronic acid (denosumab: 343 [198–804 AU] versus placebo 354 AU [76–675 AU], *P*=0.41; alendronic acid: 326 [138–813 AU] versus placebo 354 AU [76–675 AU], *P*=0.49; Figure 3A; Table I in the Data Supplement).

Mixed-effects linear regression showed no evidence of a difference between trial arms for either the denosumab-placebo model (least-squares mean: 1768 [95% CI, 1434–2101 AU] versus 1599 AU [95% CI, 1262–1936 AU], difference in means: 169 AU [95% CI, –304 to 643 AU], *P*=0.48) or the alendronic acid-placebo model (least-squares mean: 1792 [95% CI, 1452–2132 AU] versus 1596 AU [95% CI, 1253–1939 AU], difference in means: 196 AU [95% CI, –286 to 679 AU], *P*=0.42). Prespecified sensitivity analyses limited to those at least 50% (n=129) or 70% (n=118) compliant demonstrated no differences in the primary outcome (Table II in the Data Supplement). A further sensitivity analysis of the primary outcome excluding calcium scores affected by artifact in 19 participants also demonstrated no differences in the primary outcome.

### Secondary End Points

We calculated a 24-month change in peak aortic jet velocity in 136 patients (46 placebo, 46 denosumab, 44 alendronic acid) with a median time to final echocardiogram of 780 days (749–798 days), in whom the baseline peak aortic jet velocity was 3.35 m/s (2.91–3.77 m/s). There were no differences in the calculated 24-month change in peak aortic jet velocity, either between denosumab and placebo (0.49 [0.15–0.75 m/s] versus 0.33 m/s [0.12–0.59 m/s], *P*=0.21) or between alendronic acid and placebo (0.44 [0.11–0.63 m/s] versus 0.33 m/s [0.12–0.59 m/s], *P*=0.74; Figure 3B). There were no statistically significant between-group differences in calculated 24-month change in mean gradient or aortic valve area (Table III in the Data Supplement).

Aortic valve ^18^F-NaF uptake was measured in 130 participants (46 placebo, 44 alendronic acid, 40 denosumab) who underwent baseline and 12-month PET-CT (median time to scan, 418 days [406–429 days]). There were no differences in the calculated 12-month change in aortic valve mean target to background ratio either between denosumab and placebo (0.00 [–0.11 to 0.16] versus 0.03 [–0.19 to 0.15], *P*=0.87) or alendronic acid and placebo (0.06 [–0.09 to 0.21] versus 0.03 [–0.19 to 0.15], *P*=0.20; Figure 3C). There were no differences in the 12-month change in aortic valve maximum target to background ratio either between denosumab and placebo (–0.02 [–0.19 to 0.40] versus 0.01 [–0.29 to 0.31], *P*=0.61) or alendronic acid and placebo (0.12 [–0.12 to 0.40] versus 0.01 [–0.29 to 0.31], *P*=0.15). There were no differences in calculated 12-month change in mean standardized uptake value or maximum standardized uptake value between groups (Table I in the Data Supplement). Baseline aortic valve ^18^F-NaF mean target to background ratio and maximum target to background ratio correlated with the calculated 24-month change in aortic valve calcium score (*r*=0.39 and *r*=0.40, *P*<0.001 for both) and peak aortic jet velocity (*r*=0.26 and *r*=0.25, *P*=0.002 and 0.005, respectively; Figures I and II in the Data Supplement).

### Clinical and Safety Outcomes

A total of 41 participants (10 placebo, 14 denosumab, 17 alendronic acid) did not complete the final 24-month visit, 27 of whom attended at least 1 follow-up visit and therefore contributed to the primary end point (Figure 1). There were 3 deaths in each of the study arms before the final study visit. There were no differences in the median number of adverse events (placebo: 2 [1–3], alendronic acid: 2 [1–2], denosumab: 2 [1–3]) or serious adverse events (0 [0–1] for all groups; Tables IV–VI in the Data Supplement). One serious adverse event was deemed related to a study drug (alendronic acid): esophagitis leading to dysphagia ≈10 months after the study baseline visit, diagnosed on endoscopy and treated with proton pump inhibition and cessation of the study drug. No participants were unblinded during the study.

## Discussion

In this single-center, parallel-group, double-blind, randomized controlled trial, we demonstrate that treatment with denosumab or alendronic acid had no significant effect on the progression of aortic valve calcification over 24 months in asymptomatic patients with calcific aortic stenosis. We confirmed that both active trial interventions achieved inhibition of bone resorption but were unable to demonstrate an effect on the progression of aortic stenosis. We conclude that these treatments for osteoporosis do not have a major effect on the progression of aortic stenosis.

There remains a major unmet need for an effective disease-modifying noninvasive therapy in aortic stenosis. After the failure of lipid-lowering therapies,^1–3^ we hypothesized that targeting active valvular calcification might be a feasible therapeutic avenue to slow disease progression. This hypothesis was based on preclinical data demonstrating the importance of molecular triggers for calcification in the valve, as well as clinical observational data showing the close association between calcification activity measured with ^18^F-NaF PET and the subsequent progression of aortic valve calcification and stenosis severity.^12,13,22,32^ Moreover, several reports have linked increased bone resorption and osteoporosis with calcification in the aorta and aortic valve,^33,34^ and the role of active aortic valve calcification has been highlighted in previous consensus statements.^35^ The close associations between osteoporosis, bone turnover, and calcific aortic stenosis led to our repurposing of drugs used to treat osteoporosis to test this hypothesis. Our results did not reject the null hypothesis.

Given the failure to meet the primary end point, it is important to consider the potential reasons for this. First, was the trial intervention successfully applied, and did it achieve the desired pharmacological effect? Compliance was excellent in all study arms, and there were no differences between the active arms and those receiving placebos. This finding confirms that the active interventions were well tolerated in this population of patients with aortic stenosis. Furthermore, we observed the expected halving of serum C-terminal telopeptide concentrations in those receiving denosumab or alendronic acid, confirming the pharmacodynamic effect of these drugs on bone turnover and resorption in our study population. We can therefore be confident that the trial interventions were successfully administered and achieved their anticipated pharmacological effects.

We should consider whether we have failed to detect an effect of the intervention because of insensitivity of the measurements of aortic stenosis progression or a lack of power. We set out to undertake a comprehensive assessment of aortic stenosis severity and progression using 3 complementary but distinctly independent methods: aortic valve calcium scoring, Doppler echocardiography, and ^18^F-NaF PET-CT. Aortic valve calcium scoring and echocardiography are standard clinical tools used to assess disease severity,^24,36,37^ and we were able to identify and quantify disease progression across all 3 trial treatment arms using both of these methods. We observed an overall rate of hemodynamic progression that is consistent with published series and trials.^1–3,38^ In addition, we also demonstrated that baseline ^18^F-NaF PET, a measure of calcification activity, correlated with progression of both peak aortic jet velocity and aortic valve calcium score. This confirms our prior observational data demonstrating similar correlations.^22^ Thus, these techniques have assessed drug efficacy from 3 distinct but complementary approaches and found a concordant lack of effect in the 2 active trial interventions.

We acknowledge that our sample size was modest, with a preponderance of men. In addition, a proportion (13%) of patients developed a clinical indication for aortic valve replacement and did not complete the full 24-month study period. It is important to note that many of these patients still contributed to the study end points on the basis of the available imaging data at their final visit. The proportion of patients who did not complete the full study period is consistent with the severity, profile, and completion rates of previously reported randomized controlled trials of aortic stenosis therapies.^1–3^ These factors were anticipated and accounted for in our sample size calculations and statistical analysis plan. Our trial population recapitulated the same rates of disease progression, including the anticipated increase in aortic valve calcium score, which was our prespecified primary end point. We found no signal toward benefit or harm in either active treatment arm, and the 95% CIs encompassed our prespecified effect size of 40%.

Would a larger study in a population with less severe aortic stenosis demonstrate a difference? The majority (85%) of our trial population had mild or moderate disease. The natural history of aortic stenosis dictates that many years will elapse before mild aortic stenosis will become severe. Because the trial was powered for an effect size of 40%, it is possible that we failed to detect a smaller treatment effect that could delay surgery in the longer term. However, we have recently examined the contemporary use of aortic valve calcium scoring for assessing disease progression and demonstrated that modest sample sizes, not dissimilar to our present study, are needed to detect the desired effect size sought here.^24^

Given that the trial intervention achieved its intended pharmacological effect and that multiple measures of disease severity and progression did not detect a treatment effect, was the underlying hypothesis incorrect? The molecular mechanisms underlying our hypothesis have been demonstrated in preclinical models, but the direct in vivo exploration of human valvular interstitial cell osteoclastic and osteoblastic differentiation and turnover has not been established and would be very challenging to undertake. We have demonstrated previously that microcalcification activity correlates well with aortic stenosis progression, as measured by both aortic valve macrocalcification on noncontrast CT and hemodynamic stenosis on Doppler echocardiography.^22^ Severe aortic valve calcification on noncontrast CT is also strongly associated with future aortic valve replacement.^31^ We therefore continue to believe that valvular calcification remains a major pathogenetic determinant of aortic stenosis progression. However, multiple other pathways lead to calcification in the valve, with many proinflammatory mediators that initiate and promulgate disease progression. We chose denosumab and alendronic acid as established treatments for osteoporosis in an attempt to slow valvular calcification while maintaining bone health. The absence of a detectable beneficial effect on valvular calcification may suggest that the pathophysiology of calcification in aortic stenosis is independent of these pathways or that much higher doses than those used for the treatment of osteoporosis may have been needed. However, given the observed effect on markers of bone turnover, we believe the latter explanation is unlikely.

The main strength of our study is its rigorous design and the use of multiple measures of disease severity and activity to assess drug efficacy. It is the first double-blind, randomized controlled trial to test the hypothesis in question and has provided a clear answer, with concordant results across each imaging modality. However, the study is limited by its single-center design and a population skewed in ethnicity that, although representative of Scotland, may not be more widely generalizable. The underrepresentation of women in this study is an issue that we and others have encountered in previous similar trials and is clearly suboptimal.^1–3^ We did include a small number (n=11, 7%) of patients with bicuspid aortic valves. This maybe a potential confounder, because the calcific and noncalcific mechanisms underlying valve degeneration may differ from tricuspid aortic valves. We would also highlight that this trial was powered to investigate disease progression rather than clinical events. However, elective aortic valve replacement is largely based on symptom assessment and noninvasive measurements of aortic stenosis severity, and we have clearly demonstrated no treatment effect on the latter. Given the long time course of aortic stenosis, it would not have been feasible to demonstrate a difference in clinical events during the relatively short 24-month study period. However, previous trials have demonstrated concordance between measures of disease severity and subsequent large-scale clinical end point trials in aortic stenosis.^1–3^

In conclusion, we have demonstrated that denosumab and alendronic acid have no effect on the progression of aortic valve calcification or stenosis severity over 24 months in patients with asymptomatic aortic stenosis. Alternative pathways and mechanisms need to be explored to identify a disease-modifying therapy for this growing population of patients with a potentially fatal condition.

## Acknowledgments

The authors acknowledge the following parties for their contributions to the conduct of the study: Clinical Research Facility, University of Edinburgh and National Health Service Lothian; Edinburgh Imaging Facility, University of Edinburgh and National Health Service Lothian; Edinburgh Clinical Trials Unit, University of Edinburgh; Investigational Supplies Group, University of Edinburgh; Academic and Clinical Central Office for Research and Development, University of Edinburgh and National Health Service Lothian; Trial Steering Committee; British Heart Foundation; Cardiovascular Biomarker Laboratory, University of Edinburgh; Amgen Limited; Dr Fiona Strachan, University of Edinburgh; Dr Evangelos Tzolos, University of Edinburgh; Dr Soongu Kwak, Seoul National University; Dr Jacek Kwieciński, Cedars-Sinai Medical Center; Olivia Campbell, University of British Columbia; Dr Frederique Peeters, Maastricht University Medical Center; and Dr Enzo Alderete, Vall d’Hebron Hospital.

## Sources of Funding

This trial was funded by the British Heart Foundation (FS/14/78/31020). D.E.N. (CH/09/002, RG/16/10/32375, RE/18/5/34216), M.R.D. (FS/14/78/31020), and M.C.W. (FS/ICRF/20/26002) are supported by the British Heart Foundation. P.D.A. is supported by a Heart Foundation of New Zealand Senior Fellowship (1844). E.J.R.v.B. is supported by the Scottish Imaging Network (www.sinapse.ac.uk). D.E.N. is also the recipient of a Wellcome Trust Senior Investigator Award (WT103782AIA). M.R.D. is the recipient of the Sir Jules Thorn Award for Biomedical Research 2015 (15/JTA).

## Disclosures

None.

## Supplemental Materials

Data Supplement Tables I–VI

Data Supplement Figures I–II

Trial Steering Committee Members

Trial Protocol, Version 11, October 29, 2019

Protocol Version Tracker

Trial Statistical Analysis Plan, Version 3.2, April 17, 2020

## Supplementary Material


