# Supplementary material for: Effect of Denosumab or Alendronic Acid on the Progression of Aortic Stenosis: A Double-Blind Randomized Controlled Trial
Source: Circulation. 2021 Apr 29;143(25):2418–27. doi: 10.1161/CIRCULATIONAHA.121.053708 (PMC8212878; doi:10.1161/CIRCULATIONAHA.121.053708)
Supplement: Supplementary file 1 [file cir-143-2418-s001.pdf]

SUPPLEMENTARY MATERIAL

Effect of denosumab or alendronic acid on the progression of aortic stenosis

A double-blind randomized controlled trial

Tania A Pawade MD[1]*, Mhairi K Doris MD[1]*, Rong Bing MBBS[1]*, Audrey C White[1], Laura Forsyth PhD[2], Emily Evans MSc[3], Catriona Graham MSc[3], Michelle C Williams MD[1], Edwin van Beek MD[4], Alison Fletcher PhD[2], Philip D Adamson MBChB[1,5], Jack PM Andrews MD[1], Timothy RG Cartlidge MD[1], William SA Jenkins MD[1], Maaz Syed MD[1], Takeshi Fujisawa PhD[1], Christophe Lucatelli PhD[4], William Fraser MBChB[6], Stuart H Ralston MD[7], Nicholas Boon MD[1], Bernard Prendergast MD[8], David E Newby MD[1], Marc R Dweck MD[1]

[1] BHF Centre for Cardiovascular Science, University of Edinburgh, Edinburgh, UK

[2] Edinburgh Clinical Trials Unit, University of Edinburgh, Edinburgh, UK

[3] Edinburgh Clinical Research Facility, University of Edinburgh, Edinburgh, UK

[4] Edinburgh Imaging, University of Edinburgh, Edinburgh, UK

[5] Christchurch Heart Institute, University of Otago, Christchurch, New Zealand

[6] Norwich Medical School, University of East Anglia, Norwich, UK

[7] Institute of Genetics and Molecular Medicine, University of Edinburgh, UK

[8] King's College London, London, UK

SUPPLEMENTARY TABLES

Supplementary Table I
Aortic valve calcium score measurements and calculations

Timepoint	Trial allocation	N	Calcium score (AU)
**Baseline** - whole cohort	Placebo	50	1127 (617 to 2059)
	Denosumab	49	1163 (598 to 2151)
	Alendronate	51	1268 (672 to 2065)
**Baseline** - those included in primary endpoint analysis	Placebo	46	1053 (539 to 2027)
	Denosumab	46	1176 (598 to 2157)
	Alendronate	44	1076 (671 to 1851)
**12-month final visit** - those who attended 12-month but not 24-month visit	Placebo	6	1150 (830 to 2438)
	Denosumab	9	1531 (858 to 2455)
	Alendronate	10	1498 (902 to 3446)
**24-month final visit**	Placebo	40	1902 (683 to 2522)
	Denosumab	37	1502 (973 to 3465)
	Alendronate	34	1381 (848 to 2770)
**Final visit** - all who attended either 12 or 24-month	Placebo	46	1664 (686 to 2517)
	Denosumab	46	1517 (899 to 3465)
	Alendronate	44	1460 (857 to 2779)
**Calculated 24-month change** - primary endpoint*	Placebo	46	354 (76 to 675)
	Denosumab	46	343 (198 to 804)
	Alendronate	44	326 (138 to 813)

* The primary endpoint was calculated using the two green shaded rows as follows: [(final visit aortic valve calcium score – baseline visit aortic valve calcium score) / days from baseline visit to final visit] * 730. Where the participant did not attend a 24-month visit but did attend a 12-month visit, the 12-month visit was used as the final visit.
AU, Agatston Units
Median (interquartile range)

Supplementary Table II

Sensitivity analyses for the primary endpoint (24-month change in aortic valve calcium score)

**Excluding scans with artefact**		
**n=127**	**Change in calcium score (AU)**	**P value**
Placebo	292 (74 to 560)	0.16
Denosumab	339 (198 to 748)	
Placebo	292 (74 to 560)	0.36
Capsule	316 (138 to 732)	
**At least 50% compliance**		
**n=129**	**Change in calcium score (AU)**	**P value**
Placebo	364 (115 to 740)	0.55
Denosumab	343 (198 to 804)	
Placebo	364 (115 to 740)	0.68
Capsule	316 (138 to 813)	
**At least 70% compliance**		
**n=118**	**Change in calcium score (AU)**	**P value**
Placebo	357 (115 to 807)	0.78
Denosumab	329 (197 to 760)	
Placebo	357 (115 to 807)	0.87
Capsule	282 (138 to 754)	

AU, Agatston Units
Median (interquartile range)
P values - Wilcoxon rank sum test

Supplementary Table III

Change in imaging measures of disease severity

	**Placebo** n = 50	**Denosumab** n = 49	**Alendronate** n = 51	p-value
**24-Month change**				
Mean gradient (mmHg)	4 [1 to 9]	6 [1 to 12]	6 [1 to 8]	0.51
Aortic valve area (cm^2)	-0.13 [-0.29 to -0.02]	-0.14 [-0.25 to -0.08]	-0.09 [-0.25 to -0.02]	0.42
Stroke volume index (mL/m^2)	-1 [-5 to 3]	-1 [-5 to 4]	0 [-7 to 5]	0.98
**12-Month change**				
18F-NaF SUVmax	0.02 [-0.26 to 0.55]	0.10 [-0.24 to 0.53]	0.13 [-0.24 to 0.56]	0.75
18F-NaF SUVmean	0.07 [-0.22 to 0.31]	0.13 [-0.23 to 0.25]	0.08 [-0.08 to 0.33]	0.75

Missing echocardiography: n=4, 3, 7 Missing PET-CT: n=4, 5, 8.

Abbreviations: 18F-NaF, 18F-sodium fluoride; SUV, standardised uptake value; PET-CT, positron emission tomography-computed tomography.

Median [interquartile range]

P-values - Kruskal-Wallis test

Supplementary Table IV

Total number of adverse events according to trial arm and number of events per patient

	AllocatedTreatment									
	Not Randomised		Placebo		Injection		Capsules		**Total**	
	**N**	**%**	**N**	**%**	**N**	**%**	**N**	**%**	**N**	**%**
**Total**	12	100	50	100	49	100	51	100	162	100
**Number of AEs**										
0	6	50	16	32	15	31	16	31	53	33
1	5	42	19	38	16	33	18	35	58	36
2	.	.	5	10	10	20	12	24	27	17
3	1	8	6	12	5	10	5	10	17	10
4	.	.	1	2	3	6	.	.	4	2
5	.	.	2	4	.	.	.	.	2	1
7	.	.	1	2	.	.	.	.	1	1

Supplementary Table V

Total number of adverse events according to trial arm and severity

		AllocatedTreatment									
		Not Randomised		Placebo		Injection		Capsules		**Total**	
		**N**	**%**	**N**	**%**	**N**	**%**	**N**	**%**	**N**	**%**
**Total AE**		17	100	97	100	92	100	88	100	294	100
**Severity**	**Expectedness**										
Mild	Expected	.	.	4	4	6	7	4	5	14	5
	Unexpected	5	29	56	58	58	63	48	55	167	57
Moderate	Expected	.	.	5	5	2	2	8	9	15	5
	Unexpected	5	29	15	15	17	18	18	20	55	19
Severe	Expected	6	35	7	7	2	2	4	5	19	6
	Unexpected	1	6	10	10	7	8	6	7	24	8

Supplementary Table VI

Total number of serious adverse events according to trial arm and individual patients

	AllocatedTreatment									
	Not Randomised		Placebo		Injection		Capsules		**Total**	
	**N**	**%**	**N**	**%**	**N**	**%**	**N**	**%**	**N**	**%**
**Total Participants**	12	100	50	100	49	100	51	100	162	100
**Number of SAEs**										
0	3	25	29	58	31	63	29	57	92	57
1	9	75	16	32	9	18	17	33	51	31
2	.	.	3	6	7	14	2	4	12	7
3	.	.	1	2	2	4	2	4	5	3
4	.	.	1	2	.	.	1	2	2	1

SUPPLEMENTARY FIGURES AND FIGURE LEGENDS

Supplementary Figure I

Associations between disease progression and baseline aortic valve 18F-sodium fluoride uptake.

Correlations between 24-month change in aortic valve calcium score and baseline 18F-sodium fluoride maximum (A; r=0.40, p<0.001) and mean (B; r=0.39, p<0.001) target to background ratios, and between 24-month change in peak aortic jet velocity and baseline 18F-sodium fluoride maximum (C; r=0.25, p=0.005) and mean (D; r=0.26, p=0.002) target to background ratios.

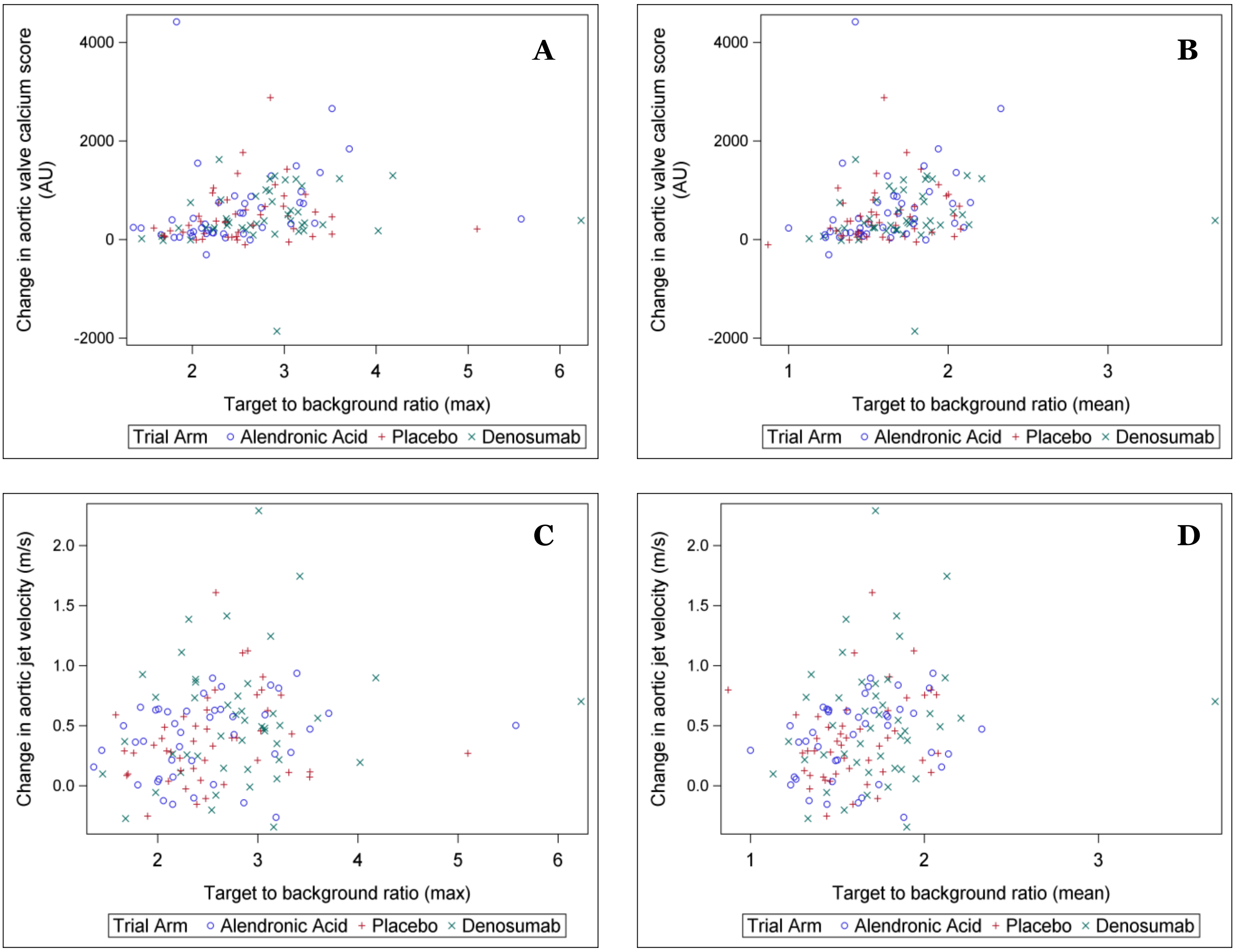

Supplementary Figure II

Example of (A) non-contrast computed tomography (CT) and aortic valve calcium (AVC) score, (B) 2-dimensional and Doppler echocardiography of peak aortic jet velocity (Vmax), and (C) CT angiography (left hand panels) and 18F-sodium fluoride maximum target to background ratio (TBR_{max}) uptake on positron emission tomography and CT angiography (right hand panels) at each study timepoint in a single representative participant. Note the enhanced anatomical definition with contrast-enhanced CT compared to echocardiography and non-contrast CT, and the increased confluence of valve calcification in regions of 18F sodium fluoride uptake.

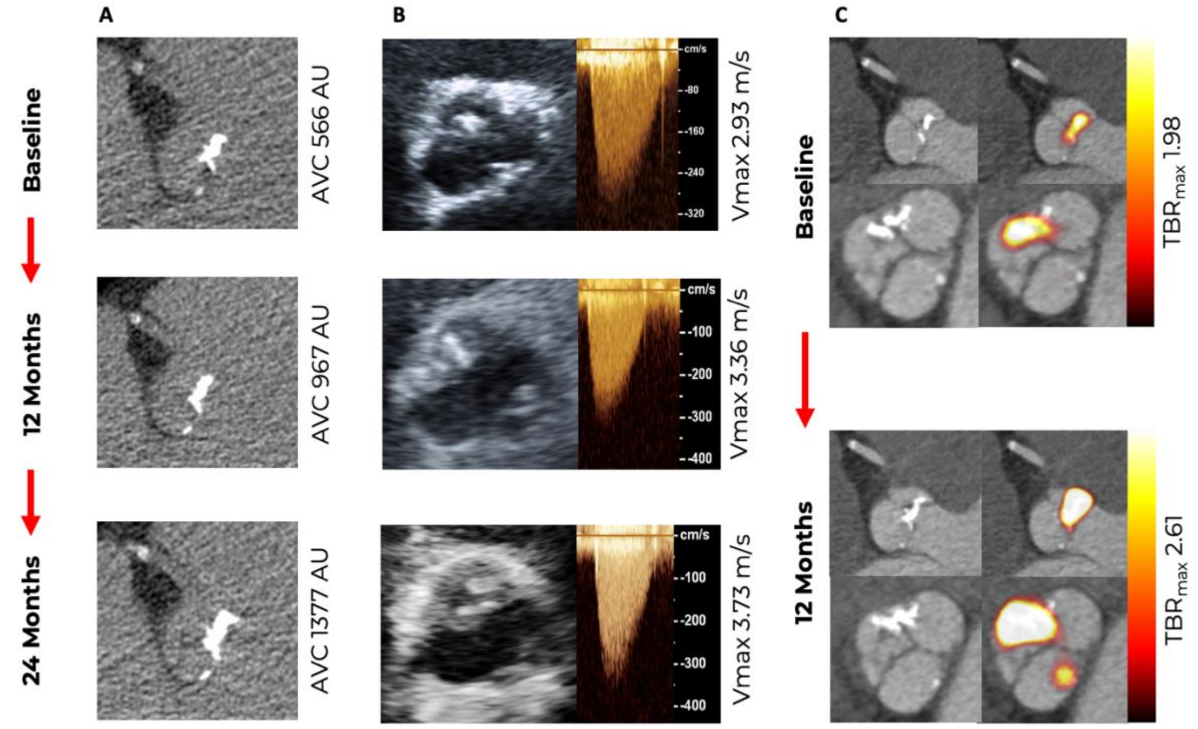

TRIAL STEERING COMMITTEE

Role	Name
Independent Chair (Cardiologist)	Professor Bernard Prendergast<br>Consultant Cardiologist<br>St Thomas' Hospital, London
Independent Member (Cardiologist)	Dr Nick Boon<br>Consultant Cardiologist<br>University of Edinburgh
Independent Member (Consultant Radiologist	Dr Giles Roditi<br>Consultant Cardiovascular Radiologist<br>NHS Greater Glasgow & Clyde
Independent Member (Lay)	Mr Bernard Hunter

Non-Independent members	
Consultant Rheumatologist	Professor Stuart Ralston<br>Molecular Medicine Centre<br>Western General Hospital<br>Edinburgh
Principal Investigator	Dr Rong Bing<br>Centre for Cardiovascular Science<br>University of Edinburgh
Supervising Investigator	Dr Marc Dweck<br>Centre for Cardiovascular Science<br>University of Edinburgh
Chief Investigator	Prof David Newby<br>Centre for Cardiovascular Science<br>University of Edinburgh
Trial Statistician	Catriona Graham<br>Lead Statistician<br>Edinburgh Clinical Research Facility<br>University of Edinburgh
Trial Manager	Dr Laura Forsyth<br>Edinburgh Clinical Trials Unit<br>University of Edinburgh
Sponsor Representative	Elizabeth Craig<br>Clinical Research Facilitator<br>Research Governance & QA Office<br>University of Edinburgh
**Observers**	
Representative of the funder (British Heart Foundation)	Dr Shannon Amoils<br>Senior Research Adviser<br>British Heart Foundation<br>London

Academic and Clinical Central Office for Research and Development

Study Protocol

Bisphosphonates and RANKL inhibition in Aortic Stenosis

The SALTIRE II Trial

Co-sponsors	University of Edinburgh & NHS Lothian<br>ACCORD<br>The Queen's Medical Research Institute<br>47 Little France Crescent<br>Edinburgh<br>EH16 4TJ
Funder	British Heart Foundation
Funding Reference Number	31020
Chief Investigator	Professor David Newby
EudraCT Number	2014-001112-19
REC Number	14/SS/0064
ClinicalTrials.gov identifier	NCT02132026
Version Number and Date	Version 11 29 October 2019

COORDINATING CENTRE

**Chief Investigator**<br><br>Professor David E Newby<br>Centre of Cardiovascular Science<br>Chancellor's Building<br>51 Little France Crescent<br>Edinburgh EH16 4SB<br><br>Tel: 0131 242 6515<br>Fax: 0131 242 6379<br>Email: d.e.newby@ed.ac.uk	**Co-sponsor Representative**<br><br>Dr Fiach O'Mahony<br>Research Governance and QA Office<br>The Queen's Medical Research Institute<br>47 Little France Crescent<br>Edinburgh<br>EH16 4TJ<br><br>Tel: (0131 242 9418<br>Fax: 0131 242 9447<br>Email: fiach.o'mahony@ed.ac.uk
**Trial Statistician**<br><br>Catriona Graham<br><br>Lead Statistician<br>Epidemiology and Statistics Core<br>Wellcome Trust Clinical Research Facility<br>University of Edinburgh<br>Western General Hospital<br>Crewe Road South<br><br>Edinburgh, EH4 2XU<br><br>Tel: 0131 537 3350<br>Fax: 0131 537 3361<br>Email: C.Graham@ed.ac.uk	**Trial Management Oversight**<br><br>Dr Laura Forsyth<br>Edinburgh Clinical Trials Unit,<br>University of Edinburgh,<br>Outpatients Building, Level 2, Room D37,<br>Western General Hospital,<br>Crewe Road,<br>Edinburgh,<br>EH4 2XU<br><br>Tel: 0131 651 9912<br><br>Email: laura.forsyth@ed.ac.uk

PARTICIPATING SITES

**Principal Investigator**<br><br>Dr Rong Bing<br>Centre of Cardiovascular Science<br>Chancellor's Building<br>51 Little France Crescent<br>Edinburgh EH16 4SB<br><br>Tel: 07510 802561<br>Email:Rong.bing@ed.ac.uk	**Supervising Investigator**<br><br>Dr Marc Dweck<br>Centre of Cardiovascular Science<br>Chancellor's Building<br>51 Little France Crescent<br>Edinburgh EH16 4SB<br><br>Tel: 07813 619 208<br>Email: mdweck@staffmail.ed.ac.uk

CONTENTS

1 INTRODUCTION 10
1.1 BACKGROUND 10
1.2 RATIONALE FOR STUDY 13
2 STUDY OBJECTIVES 13
2.1 OBJECTIVES 13
2.1.1 Primary Objective 13
2.1.2 Secondary Objectives 14
2.1.3 Safety objectives 14
2.2 ENDPOINTS 14
2.2.1 Primary Endpoint 14
2.2.2 Secondary Endpoints 14
3 STUDY DESIGN 15
4 STUDY POPULATION 17
4.1 NUMBER OF PARTICIPANTS 17
4.2 INCLUSION CRITERIA 17
4.3 EXCLUSION CRITERIA 17
4.4 CO-ENROLMENT 18
5 PARTICIPANT SELECTION AND ENROLMENT 18
5.1 IDENTIFYING PARTICIPANTS 18
5.2 CONSENTING PARTICIPANTS 18
5.3 SCREENING FOR ELIGIBILITY 18
5.4 INELIGIBLE AND NON-RECRUITED PARTICIPANTS 19
5.5 RANDOMISATION 19
5.5.1 Randomisation Procedures 19
5.5.2 Treatment Allocation 19
5.5.3 Methods to Ensure Blinding 19
5.5.4 Emergency Unblinding Procedures 19
5.5.5 Discontinuation of the Investigational Medicinal Product 20
5.5.6 Withdrawal of Study Participants 20
6 INVESTIGATIONAL MEDICINAL PRODUCT AND PLACEBO 20
6.1. STUDY DRUGS 20
6.2 STUDY DRUG 1: DENOSUMAB 20
6.2.1 Study Drug Manufacturer 20
6.2.2 Marketing Authorisation Holder 21
6.2.3 Labelling and Packaging 21
6.2.4 Storage 21
6.2.5 Summary of Product Characteristics or Investigators Brochure 21
6.2.7 Dosing Regime 21
6.3 DOSE CHANGES 21
6.4 PARTICIPANT COMPLIANCE 21
6.5 OVERDOSE 21
6.6 STUDY DRUG 2 22
6.6.1 Study Drug Manufacturer 22
6.6.2 Marketing Authorisation Holder 22
6.6.4 Storage 22

6.6.5 Summary of Product Characteristics 22
6.6.6 Placebo 2: Alendronate 22
6.8 DOSE CHANGES 22
6.9 PARTICIPANT COMPLIANCE AND ACCOUNTABILITY 22
6.10 OVERDOSE 23
6.11 OTHER MEDICATIONS 23
6.11.1 Non-Investigational Medicinal Products 23
6.11.2 Permitted Medications 23
6.11.3 Prohibited Medications 23
7 STUDY ASSESSMENTS 23
7.1 SAFETY ASSESSMENTS 23
7.1.1 Denosumab 24
7.2 STUDY ASSESSMENTS 24
7.2.1 Screening and Baseline Assessments 25
7.2.2 Follow Up Assessments 25
7.2.3 Biological Samples 26
8 DATA COLLECTION 26
9 STATISTICS AND DATA ANALYSIS 27
9.1 SAMPLE SIZE CALCULATION 27
9.2 PROPOSED ANALYSES 27
9.2.1 Description of Analysis Sets 27
9.2.2 Methods of Statistical Analysis: Main Study 27
9.2.3 Reproducibility Substudy 27
10 ADVERSE EVENTS 28
10.1 DEFINITIONS 28
10.2 IDENTIFYING AEs AND SAEs 29
10.3 RECORDING AEs AND SAEs 29
10.4 ASSESSMENT OF AEs AND SAEs 29
10.4.1 Assessment of Seriousness 29
10.4.2 Assessment of Causality 29
10.4.3 Assessment of Expectedness 30
10.4.4 Assessment of Severity 30
10.5 REPORTING OF SAEs/SARs/SUSARs 30
10.6 REGULATORY REPORTING REQUIREMENTS 31
10.7 FOLLOW UP PROCEDURES 31
11 PREGNANCY 31
12 TRIAL MANAGEMENT AND OVERSIGHT ARRANGEMENTS 31
12.1 TRIAL MANAGEMENT GROUP 31
12.2 TRIAL STEERING COMMITTEE 31
12.3 Data Monitoring Committee 32
12.4 INSPECTION OF RECORDS 32
12.5 RISK ASSESSMENT 32
12.6 BENEFIT/RISK BALANCE 32
12.6.1 Benefits 32
12.6.2 Risks 32
12.7 STUDY MONITORING AND AUDIT 33
13 GOOD CLINICAL PRACTICE 33
13.1 ETHICAL CONDUCT 33

13.2 REGULATORY COMPLIANCE 33
13.3 INVESTIGATOR RESPONSIBILITIES 33
13.3.1 Informed Consent 33
13.3.2 Study Site Staff 34
13.3.3 Data Recording 34
13.3.4 Investigator Documentation 34
13.3.5 GCP Training 34
13.3.6 Confidentiality 34
13.3.7 Data Protection 34
14 STUDY CONDUCT RESPONSIBILITIES 35
14.1 PROTOCOL AMENDMENTS 35
14.2 PROTOCOL VIOLATIONS AND DEVIATIONS 35
14.3 SERIOUS BREACH REQUIREMENTS 35
14.4 STUDY RECORD RETENTION 35
14.5 END OF STUDY 36
14.6 CONTINUATION OF DRUG FOLLOWING THE END OF STUDY 36
14.7 INSURANCE AND INDEMNITY 36
15 REPORTING, PUBLICATIONS AND NOTIFICATION OF RESULTS 36
15.1 AUTHORSHIP POLICY 36
15.2 PUBLICATION 37
15.3 PEER REVIEW 37

PROTOCOL APPROVAL

Saltire II: Bisphosphonates and RANKL Inhibition in aortic stenosis.

EudraCT number: 2014-001112-19

Signatures

Professor David Newby<br>Chief Investigator	Signature	Date
Catriona Graham<br>Trial Statistician	Signature	Date
Dr Fiach O'Mahony<br>Sponsor(s) Representative	Signature	Date
Dr Rong Bing<br>Principal Investigator	Signature	Date
Dr Marc Dweck<br>Supervising Investigator	Signature	Date

LIST OF ABBREVIATIONS

ACCORD	Academic and Clinical Central Office for Research & Development - Joint office for University of Edinburgh and NHS Lothian
AE	Adverse Event
AR	Adverse Reaction
AU	Agatston Units
BNP	Brain Natriuretic peptide
CRF	Case Report Form
CT	Computed Tomography
CTIMP	Clinical Trial of Investigational Medicinal Product
ECG	Electrocardiogram
GCP	Good Clinical Practice
IMP	Investigational Medicinal Product
ISF	Investigator Site File
ISG	Investigational Supplies Group
LDL	Low Density Lipoprotein
MHRA	Medicines and Healthcare Products Regulatory Agency
OPG	Osteoprotegerin
PET	Positron Emission Tomography
SAE	Serious Adverse Event
RANK	Receptor Activator of Nuclear Factor Kappa B
RANKL	Receptor Activator of Nuclear Factor Kappa B Ligand
SAR	Serious Adverse Reaction
SF-36	Short Form 36
SOP	Standard Operating Procedure
SUSAR	Suspected Unexpected Serious Adverse Reaction
SUV	Standard uptake volume
TBR	Tissue to Background Ratio
TMF	Trial Master File
UAR	Unexpected Adverse Reaction
18F-NaF	18F- Fluoride.
18F-FDG	18Fluorodeoxyglucose
VOI	Volume of Interest

SUMMARY

Calcific aortic stenosis is the commonest form of valve disease in the western world. It has become a major health care burden and, left untreated, is fatal. Currently, 'watchful waiting' represents the mainstay of its management with eventual aortic valve replacement triggered by the onset of symptoms. The development of an effective medical therapy has proved elusive and is a major unmet clinical need. We have demonstrated that calcification rather than inflammation is the major driver of aortic valve disease progression, and is a potential target for novel therapeutic intervention. Bisphosphonates and the Receptor Activator of Nuclear factor Kappa B Ligand (RANKL) inhibitor, denosumab, have anti-osteoporotic actions through modification of calcium homeostasis and mineralisation. These agents have the potential to reduce calcification and mineralisation of aortic valve tissue and thereby reduce or halt disease progression. We propose a double blind randomised controlled trial to establish whether alendronate or denosumab will retard or halt disease progression in patients with aortic stenosis. Outcome measures will include aortic valve calcium score by computed tomography, aortic-jet velocity determined by Doppler echocardiography, and aortic valve calcification activity by 18F-fluoride positron emission tomography. The primary end-point will be the change in aortic valve calcium score at two years. The identification of an effective medical therapy would prove a major advance in the management of patients with aortic stenosis.

1 INTRODUCTION

1.1 BACKGROUND

Pathogenesis of Aortic Stenosis

Calcific aortic stenosis has traditionally been attributed to "wear and tear" causing accelerated valvular degeneration. However there is now compelling evidence that aortic stenosis is not simply a passive process but instead the result of actively regulated inflammatory and pro-calcific processes. The initiating event is likely to resemble that of atherosclerosis whereby valvular endothelium is penetrated by lipoproteins. However, this provokes intense inflammatory activity in the valve, characterised by macrophage and T cell infiltration, and pro-inflammatory cytokine release which ultimately drives the differentiation of myofibroblasts into osteoblasts under the influence of multiple pathways including the osteoprotegerin (OPG)/Receptor Activator of Nuclear factor Kappa B (RANK)/RANK ligand (RANKL) pathway (1). It is this differentiation that is believed to be the key step in the development of aortic stenosis, with osteoblasts subsequently coordinating calcification in the valve as part of a highly regulated process, akin to new bone formation (2). Initially calcific nodules containing hydroxyapatite develop, but with time these grow and remodel so that by the end stages of the disease lamellar bone, microfractures and haemopoeitic tissue can all be identified in the valve.

1.2 Positron Emission Tomography (PET) studies in patients with aortic stenosis.

Apparent similarities in the early pathogenesis of aortic stenosis and atherosclerosis led to the hope that statins might retard disease progression in the valve as a consequence of their lipid lowering and anti-inflammatory effects. However this was refuted by the Scottish Aortic stenosis and Lipid lowering Therapy, Impact on REgression (SALTIRE) trial (3) the first double-blind randomised controlled trial of statins in patients with calcific aortic stenosis. In this trial, we demonstrated that whilst atorvastatin 80 mg daily more than halved serum LDL cholesterol concentrations and reduced C-reactive protein concentrations, it did not impact on the progression of the valvular disease process as measured by echocardiography or computed tomography calcium scoring. (4) These findings have subsequently been confirmed by two further randomised controlled trials using simvastatin combined with ezetimibe (5) and rosuvastatin (6).

As a result of the neutral findings of the statin trials in patients with aortic stenosis, we wished to reassess the underlying pathophysiology of aortic stenosis. We therefore conducted a PET study using two different radiotracers to examine the relative contributions of valvular inflammation and calcification activity in the different stages of aortic stenosis: namely 18F-fluorodeoxyglucose (18F-FDG) (7) and 18F-fluoride (18F-NaF) respectively. 18F-NaF is an established bone tracer, binding to hydroxyapatite, a key crystalline component of both bone and vascular calcification. The surface area of hydroxyapatite is much higher in regions of powdery novel calcification compared to macroscopic field calcification (where much of the hydroxyapatite is internalized and unavailable for binding) so that 18F-NaF preferentially binds to regions of novel developing calcification. It therefore acts as a marker of calcification activity, providing complementary information to the presence of established calcium on CT and demonstrating a close correlation with immunohistochemical markers of calcification activity on excised aortic valve tissue (alkaline phosphatase: r=0.65, p=0.04) (Figure 1) (8).

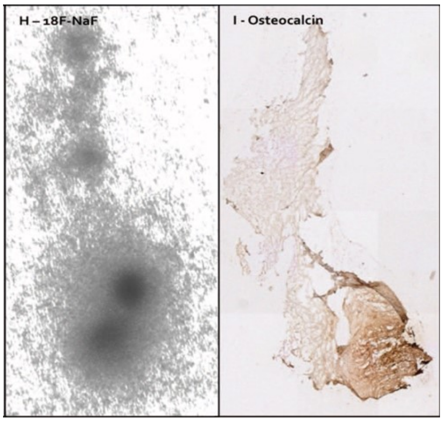

Figure 1. Aortic valve leaflet sections displaying positive immunohistochemical staining for osteocalcin (I, magnification ×4) that colocalizes to areas of maximal 18F-NaF uptake on autoradiography (H). These likely represent areas of ongoing calcification activity (9).

In 121 patients with a full range of calcific aortic valve disease, we observed 18F-NaF uptake in the aortic valve in focal areas both overlapping and adjacent to regions of established calcium on CT (Figure 2) (8). Moreover it was increased in stenotic and sclerotic aortic valves compared to control subjects, displaying a progressive rise in activity with increasing disease severity (Figure 3) (r=0.8, P<0.0001). This rise was accelerated and disproportionate to that displayed by 18F-FDG with 97% of patients with moderate aortic stenosis and all patients with severe disease displaying increased 18F-NaF activity. In contrast, uptake of 18F-FDG activity was diffuse, modest and correlated more weakly with severity (r=0.41, P<0.001) (8). Valvular uptake of the these tracers was compared to activity in regions of coincident aortic atheroma, demonstrating that whilst calcification activity was greater in the valve compared to atheroma (based upon their respective 18F-NaF uptake), the reverse was true for 18F-FDG with inflammatory activity being higher in regions of atherosclerosis (10).

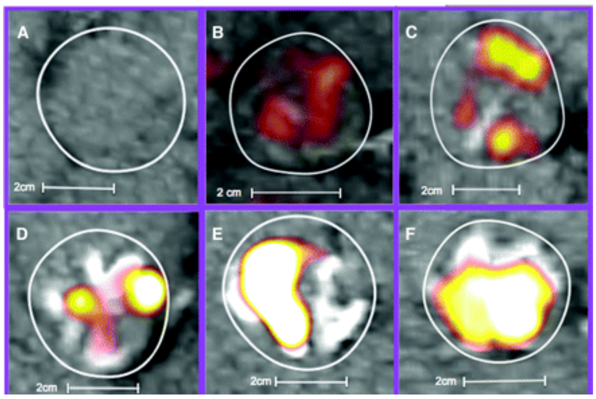

Figure 2*.* 18F-Sodium fluoride (18F-NaF) uptake in patients with aortic stenosis. 18F-NaF: Fused positron emission tomography/computed tomography scans demonstrating uptake of 18F-NaF on coaxial short-axis views of the aortic valve in patients with a normal aortic valve (A), aortic sclerosis (B), and mild (C), moderate (D), and severe aortic stenosis (E and F). White areas show regions of existing calcium, and yellow and red areas show areas of 18F-NaF uptake. Focal areas of uptake are observed in regions overlying existing calcium as well as in areas remote from it. Furthermore, areas of existing calcification are observed in the absence of overlying 18F-NaF uptake. Note the increased activity with increasing severity of valve disease. Regions of interest have been drawn around the periphery of the valve (white lines) with the use of the short-axis technique (11).

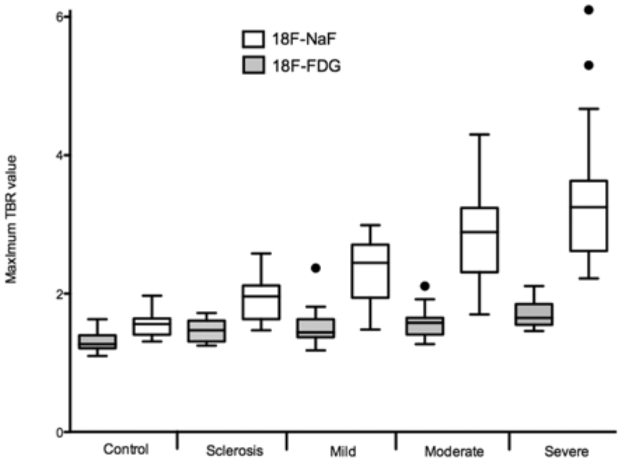

Figure 3*.* Uptake of 18F-fluorodeoxyglucose (18F-FDG) and 18F-sodium fluoride (18F-NaF) according to the severity of aortic stenosis. Box plots show the median and interquartile ranges of the tissue-to-background ratios (TBR) for 18F-NaF (white boxes) and 18F-FDG (gray boxes) with whiskers to 1.5 x interquartile range (11).

At 1 year, the baseline 18F-NaF uptake emerged as a powerful predictor of the progression in aortic valve calcification (r=0.66, P=0.002), out-performing both the current gold standard method of prediction (the baseline calcium score r=0.57, P=0.010) and 18F-FDG activity in the valve (r=0.14, P=0.55) (3). Furthermore 2-year follow up data across the cohort has confirmed a good correlation between baseline 18F-NaF uptake and the change in calcium score (r=0.78, p=<0.001). This also corresponded with both haemodynamic progression on echocardiography and adverse clinical events. Indeed even after adjustment for age and sex, baseline valvular 18F-NaF emerged as an independent predictor of aortic valve replacement and cardiovascular mortality (RR 1.19 [1.08-1.27] per 0.1 unit increment in 18F-NaF TBR, P=<0.001; unpublished data).

In summary, **calcification activity can be measured using 18F-NaF PET and appears to be the major driver of disease progression in patients with aortic stenosis.**

Bisphosphonate & RANKL Inhibition as potential treatment strategies

Regulators and mediators of calcium homeostasis are implicated in calcific aortic valve disease progression. Many cross-sectional and longitudinal studies have demonstrated a clear association between osteoporosis and an increased prevalence of both aortic stenosis and vascular calcification (12-14). There is a growing body of pre-clinical and clinical data indicating that treatments for osteoporosis, such as bisphosphonates and denosumab, can also reduce vascular calcification. These agents therefore hold considerable promise as novel therapies for aortic stenosis (15).

Bisphosponates

Bisphosphonates are inhibitors of osteoclast-mediated bone resorption, and are well tolerated in the elderly. They are widely used for the treatment of osteoporosis and interestingly have demonstrated a consistent reduction in calcification of the vasculature and the aortic valve (15-17).

Inhibition of bone resorption results in reduced release of calcium and phosphate into the circulation and increasing a reduction in the systemic availability of these pro-calcific substances (18). However bisphosphonates also exert direct anti-calcific effects on the aortic valve tissue itself. For example, they reduce the production of interleukin-1β, interleukin-6 and tumour necrosis factor-α (key inflammatory cytokines implicated in the early stages of aortic stenosis) (19) and inhibit the secretion of matrix metalloproteinases 2 and 9, which remodel the valve as aortic stenosis progresses (20, 21). Finally bisphosphonates attenuate the differentiation of aortic valve myofibroblasts into osteoblasts that, as discussed, is believed to be the key step in the development and progression of aortic stenosis. In combination these pre-clinical data offer a clear rationale for bisphosphonates as a treatment strategy for aortic stenosis that is increasingly being supported by observational clinical data. A recent analysis of 3,710 women in the Multi-Ethnic Study of Atherosclerosis (MESA) indicated that bisphosphonate use was associated with less valvular and vascular calcification in older women (22). Other studies appear to support these findings with a direct beneficial effect of these drugs on echocardiographic measures of aortic stenosis progression as well as reducing valvular calcification in patients with renal failure and amongst those with bioprosthetic valves (18, 23). Whilst encouraging, such retrospective observational studies are prone to bias, cannot assess cause-and-effect, and are confounded by the underlying effects of the osteoporosis for which these agents were prescribed. Indeed the true impact of these drugs will only become clear within the context of a randomised controlled trial.

Denosumab

The OPG/RANK/RANKL axis appears to provide further explanation for the link between osteoporosis and increased vascular calcification. Osteoprotegerin (OPG) is a decoy receptor for RANKL: a potent stimulator of osteoclast differentiation and bone resorption (24). Increased expression of RANKL and reduced levels of OPG have not only been observed in osteoporosis but also within the valves of patients with aortic stenosis (1). Moreover in rodent models, targeted genetic inactivation of OPG leads not only to high-turnover osteoporosis but also extensive vascular calcification (25). Conversely, administration of OPG to LDL-receptor deficient mice inhibits vascular calcification (26). Thus, OPG appears to determine the balance between skeletal and vascular calcification, and more specifically the development of aortic stenosis.

Denosumab is a human monoclonal antibody to RANKL that prevents its binding to RANK thereby recapitulating the actions of OPG. In a trial of 7,868 post-menopausal women with osteoporosis, denosumab (6-month 60 mg subcutaneous injections) increased bone mineral density and reduced vertebral fracture rates by 68% over a 3-year period (27). Importantly, denosumab was extremely well tolerated with very few adverse side effects and no major excess in adverse events. Given the central regulatory role that the OPG/RANK/RANKL system has in vascular and aortic valve calcification, we believe that denosumab holds considerable promise as a novel treatment for aortic stenosis, supported once more by the pre-clinical data. Indeed in a murine model of osteoporosis, denosumab demonstrated an impressive 50% reduction in aortic calcification (28). Interestingly in the same study, this reduction was closely associated with inhibited bone resorption from the skeleton, indicating that the cardiovascular effects of denosumab are, like bisphosphonates, also in part related to reduced release of calcium and phosphate from bone into the circulation.

RESEARCH HYPOTHESIS

We hypothesise that alendronate and denosumab will reduce or halt disease progression in patients with calcific aortic stenosis.

1.2 RATIONALE FOR STUDY

The rationale of this study is based on the following observations:

1) Disease progression in calcific aortic stenosis is due to active valvular calcification rather than passive ‘wear and tear’.

2) Preclinical and observational clinical data demonstrate that alendronate and denosumab are associated with inhibition of vascular and valvular calcification.

2 STUDY OBJECTIVES

2.1 OBJECTIVES

2.1.1 Primary Objective

- To determine whether administration of alendronate or denosumab can reduce or halt disease progression in patients with calcific aortic stenosis.

2.1.2 Secondary Objectives

To determine whether administration of alendronate or denosumab can:

- Reduce active calcification in the aortic valve
- Reduce aortic-jet velocity
- Reduce aortic or coronary artery calcification
- Improve bone mineral density
- Improve quality of life
- To demonstrate reproducibility of 18F-NaF PET/CT and CT calcium scoring

2.1.3 Safety objectives

To determine the safety and tolerability of alendronate or denosumab in patients with calcific aortic stenosis.

2.2 ENDPOINTS

2.2.1 Primary Endpoint

- Change in aortic valve calcium score at two years determined by computed tomography.

2.2.2 Secondary Endpoints

- Change in aortic valve 18F-NaF uptake at 12 months determined by positron emission tomography.
- Change in aortic-jet velocity at two years determined by Doppler echocardiography.
- Change in thoracic aortic and coronary artery calcium score at two years determined by computed tomography.
- Change in thoracic aorta and coronary 18F-NaF uptake at 12 months determined by positron emission tomography.
- Change in thoracic spine bone mineral density at two years determined by quantitative computed tomography.
- Change in quality of life determined by SF-EQ 36.
- Comparison of 18F-NaF PET uptake and calcium scores between the two scans in the reproducibility sub-study.

3 STUDY DESIGN

This will be a single centre double blind parallel group randomised placebo-controlled trial of alendronate and denosumab in patients with calcific aortic stenosis. The reproducibility sub-study (Table 1) will take place before the main treatment trial and will involve 20 patients who meet the eligibility criteria. These patients will not proceed into the treatment trial. Provided that reproducibility is acceptable as defined in section 9.2.3, then recruitment for the main trial will proceed as described below in table 2.

Table 1

SUBSTUDY DESIGN	Screening	Baseline	2±2 weeks
Eligibility criteria and PIS	X	X	
Consent	X		
Clinical History and Examination.		X	
Clinical biochemistry and haematology		X	X
Sampling for cardiac biomarkers (troponin, BNP, Galectin)		X	X
18F-fluoride PET		X	X
CT Calcium Scoring		X	X
CT angiography		X	X
AE/SAE reporting	X	X	X

Table 2

MAIN STUDY DESIGN	Screening *	Baseline	Random-isation 6 ± 2 weeks	2 Weeks (±2 days)	6 month (±14 days)	12 months (±14 days)	18 months (±14 days)	24 months (±14 days)
Eligibility criteria and PIS	X	X						
Clinical history and examination		X			X	X	X	X
Consent	X							
Short Form - 36 + 6 minute walk test		X						X
Concomitant medications	X	X			X	X	X	X
Full blood count, urea, creatinine, eGFR, electrolytes including calcium, liver function tests, cholesterol, glucose		X			X	X	X	X
Whole blood sampling for genetic analysis		X						
Serum vitamin D measurement		X						
Sampling for biomarkers (troponin, BNP, Galectin, c telopeptide.)		X			X	X	X	X
12-Lead electrocardiogram		X			X	X	X	X
18F-fluoride PET		X				X		
CT Calcium Scoring		X				X		X
CT angiography		X				X		
Echocardiography	X**	X**			X	X	X	X
AE/SAE reporting	X	X	X		X	X	X	X
Treatment allocation			X					
Compliance check for those in tablet arm.					X	X	X	X
Dispensing the IMP			X		X	X	X	
Telephone call to ensure dosing regimen of Alendronic acid is followed				X				
Telephone call for Denosumab arm to assess for symptoms of hypocalcaemia.				X				

* Where possible screening and baseline visits will be combined.
** Echocardiography will be performed at either screening or baseline

The main study will be a randomised double blind placebo controlled trial to ensure a rigorous study design and the avoidance of systematic biases of outcome measures.

All recruited patients will undergo baseline clinical assessment including routine haematological and biochemical profiling, echocardiography (if required), computed tomography calcium scoring, computed tomography, aortic angiography and 18F-NaF positron emission tomography scanning. A medical doctor within the research team will confirm eligibility by wet signature on the relevant case record form before any patient is randomised to a treatment arm.

4 STUDY POPULATION

4.1 NUMBER OF PARTICIPANTS

The first 20 patients who meet the inclusion and exclusion criteria for the main study will be recruited to undergo 18F-NaF PET-CT reproducibility studies and blood sampling **only**.

We will then recruit 150 patients with calcific aortic stenosis for the SALTIRE II trial. Recruitment is anticipated to take one year with follow up lasting 2 years.

4.2 INCLUSION CRITERIA

For inclusion in the study, participants should fulfil the following criteria

1) age >50 years
2) peak aortic jet velocity of >2.5 m/s on Doppler echocardiography
3) grade 2-4 calcification of the aortic valve on echocardiography

4.3 EXCLUSION CRITERIA

Participants will not enter the study if any of the following exclusion criteria are fulfilled

1) Anticipated or planned aortic valve surgery in the next 6 months,
2) Life expectancy <2 years,
3) Inability to undergo scanning
4) Treatment for osteoporosis with bisphosphonates or denosumab.
5) Long-term corticosteroid use.
6) Abnormalities of the oesophagus or conditions, which delay oesophageal/gastric emptying.
7) Inability to sit or stand for at least 30 minutes.
8) Known allergy or intolerance to alendronate or denosumab, or any of their excipients
9) Hypocalcaemia
10) Regular calcium supplementation
11) Dental extraction within 6 months
12) History of osteonecrosis of the jaw
13) Major or untreated cancers
14) Poor dental hygiene
15) Women of child-bearing potential who have experienced menarche, are pre-menopausal, have not been sterilised or who are currently pregnant.
16) Women who are breastfeeding
17) Renal failure (estimated glomerular filtration rate of <30 mL/min)
18) Allergy or contraindication to iodinated contrast

19) Inability or unwilling to give informed consent
20) Likelihood of non-compliance to treatment allocation or study protocol.

4.4 CO-ENROLMENT

Co-enrolment with non-CTIMP studies will be considered on a case-by-case basis and will consider additional radiation and the extra burden being placed on the study participants. Similarly co-enrolment with other CTIMPs will also be considered on a case-by-case basis, taking into consideration the burden and potential risks placed on the participant and the interactions between the IMPs involved. Arrangements for co-enrolment with another CTIMP will be bound by a written agreement between the Chief Investigator and Co-Sponsors of both/all CTIMPs implicated.

5 PARTICIPANT SELECTION AND ENROLMENT

5.1 IDENTIFYING PARTICIPANTS

Patients will be predominantly identified and recruited from the cardiology outpatient clinics of the Edinburgh Heart Centre with the aid of our echocardiography database. All patients will be approached for study recruitment by their usual care team. Patients will be provided with a Patient Information Sheet and given an opportunity to ask questions about participation in the trial.

In addition, other hospitals in Scotland will be set up as Participant Identification Centres (PICs). The PICs will identify patients from their cardiology databases and cardiology outpatient clinics and a member of the patients usual care team will provide the patient either in person or by post with a Patient Information Sheet. Those patients who are interested in participating in the study or obtaining further details will be asked to contact the research team in Edinburgh to discuss the study further.

5.2 CONSENTING PARTICIPANTS

After 2 days, patients willing to participate in the trials will be asked to attend a screening visit at the Clinical Research Facility. Written informed consent will be obtained by a suitably qualified member of the research team before any study related procedures are performed.

5.3 SCREENING FOR ELIGIBILITY

The patient's usual care team will identify potential participants. If eligibility cannot be confirmed based on pre-existing clinical investigations (i.e. renal function and calcium levels are not available within the past 6 months) blood tests will be obtained to assess these variables once informed consent has been obtained. An echocardiogram will also be performed during the screening visit if this is required to confirm eligibility. Those patients who have an echocardiogram performed during the screening visit will not need echocardiography performed at their baseline visit.

Once a patient has agreed to participate and is deemed eligible, they will be invited to attend the baseline visit. Pregnancy testing will be performed when necessary although this is anticipated to be extremely infrequent.

Baseline assessments for the main trial will include clinical history and examination, review of patient records to confirm study eligibility and record clinical profile, standard clinical biochemical and haematological variables 12-lead electrocardiogram and echocardiography, if required.

In the event that recent bloods are available and it is considered probable that a participant will be eligible after review of the medical notes, the screening and baseline visits will be combined. Screening and baseline visits will also be combined, in the absence of available bloods, for those participants with poor mobility and those travelling from long distances. In

these participants, renal function and serum calcium levels will be checked prior to any baseline assessments being performed.

5.4 INELIGIBLE AND NON-RECRUITED PARTICIPANTS

Ineligible and non-recruited patients will receive standard medical care. An anonymised log will be kept of patients who were screened for the study and subsequently found to be ineligible or not recruited.

5.5 RANDOMISATION

5.5.1 Randomisation Procedures

A web-based computer-generated randomisation process will be used to ensure allocation concealment and reduce bias, with minimisation techniques employed to ensure balancing of key variables: age, sex, and the baseline aortic valve calcium score. Patients will be allocated to one of four groups [denosumab injection, placebo injection, alendronate capsule or placebo capsule in a 2:1:2:1 ratio using a minimisation algorithm that incorporates a random component. Factors used in the minimisation will be age [<73 and ≥73 years], sex, presence or absence of a bicuspid valve and baseline aortic valve calcium scores (‘≤1607, >1607 Agatston Units) (a marker of disease severity).

5.5.2 Treatment Allocation

Seventy-five patients will be randomised (2:1) to either subcutaneous denosumab 60 mg (n=50) or matched placebo (n=25) every 6 months; and a further 75 will be randomised (2:1) to oral alendronate 70 mg (n=50) or matched placebo (n=25) once weekly. Treatment will be allocated by a dedicated web-based computer randomisation in a ratio of 2:1:2:1 for alendronate:alendronate placebo:denosumab: denosumab placebo

5.5.3 Methods to Ensure Blinding

The inactive placebo comparator for alendronic acid will be manufactured by the Investigational Supplies Group (ISG) of the University of Edinburgh, so that it matches and is indistinguishable from the overencapsulated alendronate. The Investigational Medicinal Product will be presented in numbered packages in collaboration with the Investigational Supplies Group.

Denosumab is manufactured as a prefilled syringe, and the denosumab placebo will be an injection of 0.9% saline. The pre-administration preparation and administration will be performed by a specific group of nominated nurses at the clinical research facility who will remain unblinded throughout the course of the study. Both the denosumab and saline will be covered in foil in order for the research team and patients to remain blinded. Nurses being asked to conduct investigations on behalf of the research team will also remain blinded.

Half the study population will receive alendronate/placebo capsules once weekly and the other half will receive 6 monthly injections of denosumab/placebo. Therefore, participants and investigators will know which limb of the study they are participating in but will not know whether they are on active or placebo medication.

5.5.4 Emergency Unblinding Procedures

Unblinding may take place in situations where the safe management of the participant’s medical condition necessitates knowledge of the study medication by the person(s) responsible for the participant’s care. 24 hour unblinding is available and will be performed by the Principal Investigator via the trial database. The reason for unblinding, the person

responsible for performing the unblinding and the participant number will be recorded in the database.

5.5.5 Discontinuation of the Investigational Medicinal Product

The investigational product will be discontinued under the following circumstances:

1. At the request of the patient or if the patient withdraws from the study.
2. By the investigator or the responsible clinician if this was felt to be in the best interests of the patient
3. On completion of the study.

5.5.6 Withdrawal of Study Participants

Participants are free to withdraw from the study at any point or a participant can be withdrawn by the investigator. If withdrawal occurs, the reasons for withdrawal will be documented where available in the participant's case record form.

If a patient who has consented for the study states that they wish to be withdrawn from the study before randomisation, they will be withdrawn from the study and another participant will be recruited to replace them.

6 INVESTIGATIONAL MEDICINAL PRODUCT AND PLACEBO

6.1. STUDY DRUGS

There are two IMPs for the study: denosumab and alendronate.

6.2 STUDY DRUG 1: DENOSUMAB

Denosumab (Prolia, Amgen) 60 mg solution for injection in a pre-filled syringe. The solution is a clear, colourless to slightly yellow solution.

Investigational product	Dosage form and strength	Manufacturer
Denosumab (Prolia)	60 mg/mL injection	Amgen

6.2.1 Study Drug Manufacturer

Amgen

Manufacturing site:
Amgen Technology Ireland (ADL)
Pottery Road
Dun Laoghaire
Co Dublin
Ireland
QC testing is on site.

Packing site:
Amgen Europe B.V.
Minervum 7061
NL-4817 ZK Breda
The Netherlands
QA is managed on site.

6.2.2 Marketing Authorisation Holder

Amgen
Amgen Europe B.V.
Minervum 7061
NL-4817 ZK Breda
The Netherlands

MA number: EU/1/10/618/003

6.2.3 Labelling and Packaging

Study medication will be provided in commercial packs and labelled with a clinical trial label for the purpose of this trial including the study title, trial participant number and study contact.

The Investigational Supplies Group (ISG), University of Edinburgh will be responsible for clinical trial labelling.

6.2.4 Storage

Denosumab needs to be stored in a refrigerator (2–8°C). Once removed from the refrigerator, Denosumab must be used within a 30-day period.

6.2.5 Summary of Product Characteristics

The Summary of Product Characteristics (SPC) can be found in the Investigator Site File or the Trial Master File.

6.2.6 Placebo 1: Denosumab.

The placebo will be 1 mL 0.9% saline injections and will be administered subcutaneously 6 monthly for 2 years.

6.2.7 Dosing Regime

6-monthly 60 mg subcutaneous injections for 2 years (four doses in total).

6.3 DOSE CHANGES

No alteration in the planned dosing regime will be allowed

6.4 PARTICIPANT COMPLIANCE

The research nurses will be administrating the subcutaneous injections and therefore compliance will be ensured.

6.5 OVERDOSE

There is no experience with overdose in clinical studies. Denosumab has been administered in clinical studies using doses up to 180 mg every 4 weeks (cumulative doses up to 1,080 mg over 6 months), and no additional adverse reactions were observed.

6.6 STUDY DRUG 2

Investigational product	Dosage form and strength	Manufacturer
Alendronic Acid	70 mg tablets.	TEVA UK Limited

6.6.1 Study Drug Manufacturer

TEVA UK Limited
Brampton Road, Hampden Park
Eastbourne, BN22 9AG
England

Alendronic acid will also be packaged and subject to Quality Control checks at the above site.

6.6.2 Marketing Authorisation Holder

TEVA UK Limited
Brampton Road, Hampden Park
Eastbourne, BN22 9AG
England

The marketing authorisation number is PL 00289/1956.

6.6.3 Labelling and Packaging

Overencapsulation of alendronate capsules (with lactose monohydrate back fill), bottling and clinical trial labelling will be performed by ISG.

6.6.4 Storage

The study drugs will be stored within the pharmacy at the Edinburgh Royal Infirmary, at ambient temperature (15-25°C).

6.6.5 Summary of Product Characteristics

The Summary of Product Characteristics (SPC) can be found in the Investigator Site File or the Trial Master File.

6.6.6 Placebo 2: Alendronate

Manufacture, packaging into bottles and clinical trial labelling of hard gelatin placebo capsules containing Lactose Monohydrate will be performed by ISG.

6.7 DOSING REGIME

Once weekly capsule 70mg for 2 years.

6.8 DOSE CHANGES

No alteration in the planned dosing regime will be allowed. If a participant misses a dose they should take one tablet on the morning after they remembered but cannot take two tablets on the same day.

6.9 PARTICIPANT COMPLIANCE AND ACCOUNTABILITY

The nurses at the Clinical Research Facility will assess treatment compliance at attendance of each of the study visits by interview and pill count.

6.10 OVERDOSE

No specific information is available on the treatment of overdosage with alendronic acid. Hypocalcemia, hypophosphatemia, and upper gastrointestinal adverse events, such as upset stomach, heartburn, oesophagitis, gastritis, or ulcer, may result from oral overdosage. Milk or antacids should be given to bind alendronate. Due to the risk of oesophageal irritation, vomiting should not be induced and the patient should remain fully upright. Dialysis would not be beneficial.

6.11 OTHER MEDICATIONS

6.11.1 Non-Investigational Medicinal Products

18F-Sodium Fluoride (18F-NaF) will be used for the PET CT scans. This radiotracer will be manufactured in the Clinical Research Imaging Centre using MHRA licensed facilities for the purpose of this study.

At the time of positron emission tomography and computed tomography coronary angiography, patients may receive oral and/or intravenous beta-blockade, such as metoprolol 5-50 mg, to slow the heart rate to below 65 beats per minute to maximise image quality and reduce radiation exposure.

Permitted Medications

All patients will be maintained on their regular medications. Patients who are already on medications for osteoporosis including calcium supplementation are to be excluded from the trial. Following completion of the study, the trial medications will be discontinued.

6.11.2 Prohibited Medications

There are no prohibited medications other than those stipulated in the exclusion criteria. Patients should not be receiving any treatment for osteoporosis in the form of bisphosphonates, denosumab or calcium supplementation. If a participant is commenced on denosumab or bisphosphonates for the management of osteoporosis during the course of the study, they will be no longer receive the study drugs, but will continue to be followed up and retained in the analyses. Participants may be commenced on calcium supplementation by the investigators in the event of hypocalcaemia as discussed above. Under these circumstances the patient will be retained in the study.

7 STUDY ASSESSMENTS

Trial participants for the reproducibility study will attend for 3 visits; screening, baseline and a single follow up. Those participating in the main trial will undergo a maximum of 7 study visits: screening visit, baseline and randomisation visits and then follow up visits will take place at 6, 12, 18 and 24 months. Time windows to capture follow-up data are detailed in section 3.

7.1 SAFETY ASSESSMENTS

7.1.1 Denosumab

Denosumab is licensed for the treatment of osteoporosis and its side-effect profile is well documented. This includes musculoskeletal pain, constipation, urinary tract infection, upper respiratory tract infection, pain in extremity, sciatica, cataracts, rash; less commonly diverticulitis, cellulitis, ear infection, eczema; rarely osteonecrosis of the jaw; and very rarely hypocalcaemia. In the FREEDOM trial, cellulitis was the only side-effect that was more

common than placebo in patients treated with denosumab. This will be a particular focus of the safety reporting of the trial.

Osteonecrosis of the jaw has been reported in association with denosumab (rarely) and alendronic acid. Risk factors for this rare but serious adverse event are poor oral hygiene, dental extractions, and co-morbid disorders, especially patients with cancer or receiving corticosteroids.

We will refer patients to a dentist before study enrolment if poor dental hygiene is identified, with their permission we will confirm their subsequent suitability to enter the trial by contacting their dentist. Patients will not be recruited within 6 months of a dental extraction (if the patient cannot confirm this, with their permission we will contact their dentist). We will exclude patients with major or untreated cancers. Women of child bearing potential who have experienced menarche, are pre-menopausal, have not been sterilised or who are currently pregnant and/or breastfeeding will be excluded from the study.

We will ensure that patients are not hypocalcaemic or vitamin D deficient and where necessary this will be addressed appropriately by oral supplementation. Calcium levels will be measured prior to each dose of denosumab which will only be administered if calcium levels are above the lower limit of normal i.e the participant is not hypocalcaemic The exclusion criteria have been designed specifically to exclude those patients who are at risk of developing severe, symptomatic hypocalcaemia (patients with severe renal impairment defined as GFR <30 and those receiving dialysis). The risk of hypocalcaemia is greatest after the first dose therefore all participants in the denosumab arm will receive a 2 week phone call to assess the development of symptoms. These include muscle spasm, seizures, numbness/tingling, irritability or confusion. Participants will also be advised to be vigilant for these symptoms of hypocalcaemia. If there is a concern that a participant has developed hypocalcaemia, their calcium levels will be measured and treated if appropriate. Continuation with the study drug would then be reviewed by the Trial Steering Committee.

7.1.2 Alendronate

Alendronate is a bisophosphonate that is primarily used in the treatment of post-menopausal osteoporosis. We will exclude patients who have contraindications to alendronate such as abnormalities of the oesophagus and other factors which delay oesophageal emptying such as stricture or achalasia, inability to stand or sit upright for at least 30 minutes, hypersensitivity to alendronate or to any of the excipients or hypocalcaemia.

The main side effect is upper gastrointestinal symptoms specifically oesophagitis, oesophageal ulcers and erosions. It is also associated with gastric and duodenal ulcers. These side-effects are more commonly seen in patients who fail to take the medication according to instructions. Therefore careful emphasis will be placed on instructing patients how best to take the medication. Whilst no increased risk of upper gastrointestinal complications has been reported in clinical trials, this will be a focus for adverse event reporting. Other side-effects of bisphosphonates include muscle pains which are rarely severe or incapacitating.

7.2 STUDY ASSESSMENTS

Reproducibility Study

Twenty patients who meet the inclusion and exclusion criteria will solely undergo the substudy (18F-NaF PET/CT imaging, CT angiography and CT calcium scoring). Screening of clinical records for eligibility will be performed after written consent is obtained. If eligibility cannot be confirmed based on pre-existing clinical investigations then blood tests will be obtained to assess these variables. Two 18F-fluoride PET scans will be performed within two weeks of each other to demonstrate scan-rescan reproducibility. A clinical history and

examination will be performed at the baseline visit and blood samples will be obtained on both visits for biochemical and haematological profiling, cardiac biomarkers (troponin, BNP, Galectin) and storage for future studies, however they will not participate further with the trial.

Main Study

7.2.1 Screening and Baseline Assessments

Screening of clinical records for eligibility will be performed after written consent is obtained. If eligibility cannot be confirmed based on pre-existing clinical investigations (i.e. calcium levels and renal function have not been assessed within the past 6 months) blood tests will be obtained to assess these variables. In the event that recent bloods are available and it is considered probable that a participant will be eligible after review of the medical notes, the screening and baseline visits will be combined. Screening and baseline visits will also be combined, in the absence of available bloods, for those participants with poor mobility and those travelling from long distances. In these participants, renal function and serum calcium levels will be checked prior to any baseline assessments being performed.

An echocardiogram will be performed during the screening visit for those participants where it is required to confirm eligibility. Those participants who have an echocardiogram performed at the screening visit will not need to have echocardiography performed at their baseline visit.

Baseline assessments will include clinical history and examination, review of patient records to confirm study eligibility and record clinical profile, standard clinical biochemical and haematological variables, 12-lead electrocardiogram, echocardiography (if required), 18F-fluoride positron emission tomography, computed tomography angiography, aortic valve computed tomography calcium scoring, and storage of plasma and serum.

7.2.2 Follow Up Assessments

Clinical Assessment
At 6, 12, 18 and 24 months, participants will undergo repeat clinical assessment, blood sampling and 12-lead electrocardiogram. Compliance will be recorded by patient history and tablet count. Patients allocated to the tablet arm of the study will receive a two week phone call to ensure that tablets are being taken as instructed (once weekly and not once daily). Those allocated to the injection arm will receive a two week phone call after the first dose to assess for symptoms of hypocalcaemia. At baseline and 24 months, patients will be asked to complete a 6 minute walk test and a SF - 36 Questionnaire.

Echocardiography: Aortic stenosis severity will be assessed at baseline and every 6 months by a single, dedicated research ultrasonographer to assess aortic stenosis severity using the peak aortic valve velocity, the mean gradient, aortic valve area and aortic valve calcification score.

Computed Tomography Calcium Scoring
Aortic stenosis severity will be assessed at baseline, 12 months and 2 years by computed tomography. CT Calcium Scoring of the aortic valve will be performed using the same protocol and scanner (Biograph 128, Siemens) at each time point.

18-NaF PET/CT imaging
Study scans will be performed at baseline and at 12 months and will take place in the Clinical Research Imaging Centre.

18F-NaF PET/CT imaging will be performed using a combined PET and 128-multidetector CT scanner (Biograph mCT, Siemens) approximately 60 min after administration of 18F-NaF (125 MBq). Recent dynamic imaging data have demonstrated that this time point offers the

optimal contrast between uptake in vascular tissue and the blood pool. Those with a heart rate of >65 /min will be given beta-blockade. PET acquisition will be ECG gated and performed using a single 30-min bed position centred on the valve in three-dimensional mode. PET images will be reconstructed in diastole (50-75% of the RR interval) with correction applied for attenuation (assessed on CT), dead time, scatter and random coincidences. Finally CT calcium scoring and an ECG-gated contrast-enhanced CT angiogram will be performed of the aortic valve to allow co-registration and accurate co-localisation of the PET signal to individual valve leaflets. This protocol was used in our recent study examining 18F-NaF uptake in the coronary arteries: total radiation exposure of 8-9 mSv (similar to an invasive coronary angiogram) (7). If, however, a participant develops a contraindication to the contrast agent prior to the 12 month PET-CT scan then the CT scan can be performed without contrast. To estimate aortic valve 18F-NaF uptake, volumes of interest (VOI) will be drawn around the aortic valve and its annulus, and used to calculate mean and maximum standardized uptake values (SUV). Tissue to background ratios (TBR) will be calculated after correcting SUV values for blood-pool activity in the superior vena cava. 18F-NaF uptake will also be measured in the aorta, coronary arteries and skeleton to assess the impact of drug therapy on calcification activity in a range of tissues and to provide additional mechanistic information (29, 30).

7.2.3 Biological Samples

Blood samples will be collected at baseline and 6, 12, 18 and 24 months. A blood sample will also be collected at screening if renal function and calcium levels are not available within the last 6 months. Blood samples will be used for routine clinical biochemistry and haematology (including full blood count, urea, creatinine and electrolytes, calcium, liver function tests, total cholesterol and glucose). Blood will also be used for cardiac biomarker measurement which will be troponin, galectin and brain natriuretic peptide. We will also measure serum C-Telopeptide. This is a marker of bone turnover and will be required to confirm that the study drugs are effectively inhibiting bone turnover. A serum vitamin D measurement will also be taken at the baseline visit. If a patient develops hypocalcaemia during the study the vitamin D levels will be remeasured. Remaining blood will be stored for future use. Approximately 30 mL of blood will be obtained on each visit. Finally at the baseline visit, blood will also be taken for genetic analysis in order to establish if certain polymorphisms in genes encoding RANK/RANKL or OPG are associated with aortic stenosis.

Blood samples for safety and routine profiling will be processed in the Royal Infirmary, Edinburgh and Clinical Research Facility. Serum for biomarkers will be stored in the Clinical Research Facility. Blood samples will be processed (plasma and serum) and stored at -80°C for later analysis. All samples will be stored in locked secure freezers and in compliance with the sponsor’s tissue governance policies.

All samples will be retained unless consent is withdrawn by the participant who specifically requests that their samples are destroyed.

8 DATA COLLECTION

All trial data will be initially recorded on paper case record form (CRF). The CRF will hold all study information and will be kept in secure facilities. This data will then be entered into a database created in association with the Edinburgh Clinical Trials Unit.

There will be no personally identifiable data on the CRF however it will include the trial participant number, baseline demographics, past medical history, current medication, symptomatic status and ECG findings.

9 STATISTICS AND DATA ANALYSIS

9.1 SAMPLE SIZE CALCULATION

Main Study

In our previous study, we have baseline and 2-year calcium scores in participants. This study included participants with mild disease so would not meet the inclusion criteria of the present study. As such, we have taken patients with aortic valve calcium scores of ≥400. The change in aortic valve calcium score appears to be skewed [median (interquartile range) 565 (190.5, 910.0)] so a log-transformation has been applied to the data resulting in a mean (sd) of 6.103 (0.867). To detect a difference of 40% in the back transformed mean aortic valve calcium score [i.e 447.2 to 268.3] would need a sample size of 47 participants per group assuming a two-sided, two-sample test with 5% level of significance and 80% power.

This calculation assumes that it is appropriate to combine both of the placebo arms to compare against denosumab and also alendronate. It also does not take into account multiple comparisons and to account for potential drop-outs we have increased the total sample size to 150.

Reproducibility Study

For the reproducibility study, we have selected 20 patients based on previous PET-CT reproducibility studies using 18F-FDG (4) as well as the variance observed in our own baseline and 2-year scan results (see above).

9.2 PROPOSED ANALYSES

9.2.1 Description of Analysis Sets

Main Study

For the purpose of analysis, we will retain participants in the treatment groups to which they were originally assigned irrespective of the treatment actually received with the exception of adverse event data which will be presented by treatment received. For patients who undergo aortic valve replacement surgery within 24 months of randomisation, we will bring forward and perform their 12-month or 24-month visit prior to discontinuation of study medication and the conduct of surgery. These patients will be retained within the analysis. All patients withdrawn from the study that have unresolved (S)AEs will be monitored until resolution of the event or until no longer medically indicated.

9.2.2 Methods of Statistical Analysis: Main Study

Descriptive analysis of participant characteristics will be presented split by treatment allocation. These analyses will be performed comparing alendronate versus placebo and separately for denosumab versus placebo.

Baseline to two-year change will be analysed using two sample *t*-tests or non-parametric equivalent as appropriate: aortic valve score, aortic valve NaF uptake, aortic jet velocity, thoracic calcium score, coronary calcium score, bone mineral density and SF-36. It is known that calcium scores tend to follow a skewed distribution and often a log-transformation is sufficient to result in normally distributed data, before using a non-parametric method appropriate transformations will be explored and utilised if appropriate.

9.2.3 Reproducibility Substudy

With respect to 18F-NaF, the PET tracer uptake is quoted as a tissue to background ratio (TBR). An expected treatment effect of 40% should result in a (TBR) change of 0.5. This is based on the difference in the activity between patients with moderate disease compared to the controls from our previous work: moderate (Max TBR) 2.89 SD 2.31-3.24, control (Max TBR) 1.56 SD 1.41-1.64. Results in a similar reproducibility study for FDG showed the width

of the 95% limits of agreement of ± 0.1-0.2 with respect to the max TBR in 11 patients. These data will be presented and analysed using a Bland Altman Analysis. If it is possible to demonstrate reproducibility by the 95% limits of agreement within ± 0.2 TBR within the first 15 patients we will proceed to the main study. If the 95% limits of agreement are ± 0.4 TBR then we will proceed to recruit 20 patients. If we are unable to demonstrate reproducibility, i.e. 95% limitis of agreement greater than ± 0.4 TBR in 20 patients then we will review the protocol of the main study before proceeding. These decisions to proceed with the main trial will be made by the Trial Steering Committee.

10 ADVERSE EVENTS

The Investigator, or another suitably qualified physician in the research team, is responsible for the detection and documentation of events meeting the criteria and definitions detailed below.

Full details of contraindications and side effects that have been reported following administration of the IMP can be found in the relevant Summary of Product Characteristics (SmPC).

Participants will be instructed to contact their Investigator at any time after consenting to join the trial if any symptoms develop.

Pre-existing Medical Conditions and Underlying Symptoms:

Medical occurrences or symptoms which are expected due to the participant's underlying disease or pre-existing condition(s) (i.e. existed before informed consent) should be recorded as medical history and only recorded as adverse events if medically judged to have worsened during participation in the trial.

In the case of an AE, the Investigator should initiate the appropriate treatment according to their medical judgment.

10.1 DEFINITIONS

An **adverse event** (AE) is any untoward medical occurrence in a clinical trial participant which does not necessarily have a causal relationship with an investigational medicinal product (IMP).

An **adverse reaction** (AR) is any untoward and unintended response to an IMP which is related to any dose administered to that participant.

A **serious adverse event** (SAE), **serious adverse reaction** (SAR). Any AE or AR that at any dose:

- results in death of the clinical trial participant;
  is life threatening*;
- requires in-patient hospitalisation^ or prolongation of existing hospitalisation;
- results in persistent or significant disability or incapacity;
- consists of a congenital anomaly or birth defect;
- results in any other significant medical event not meeting the criteria above.

*Life-threatening in the definition of an SAE or SAR refers to an event where the participant was at risk of death at the time of the event. It does not refer to an event, which hypothetically might have caused death if it were more severe.

^Any hospitalisation that was planned prior to randomisation will not meet SAE criteria. Any hospitalisation that is planned post randomisation will meet the SAE criteria.

A suspected unexpected serious adverse reaction (SUSAR) is any AR that is classified as serious and is suspected to be caused by the IMP, that it is not consistent with the information about the IMP in the Summary of Product Characteristics (SmPC) or Investigators Brochure.

10.2 IDENTIFYING AEs AND SAEs

All AEs experienced post consent and indicate the worsening of a pre-existing condition and SAEs will be recorded from the time a participant signs the consent form to take part in the study.

Participants will be asked about the occurrence of AEs/SAEs at every visit during the study. Open-ended and non-leading verbal questioning of the participant will be used to enquire about AE/SAE occurrence. Participants will also be asked if they have been admitted to hospital, had any accidents, used any new medicines or changed concomitant medication regimens. If there is any doubt as to whether a clinical observation is an AE, the event will be recorded.

AEs and SAEs may also be identified via information from support departments e.g. laboratories.

10.3 RECORDING AEs AND SAEs

When an AE/SAE occurs, it is the responsibility of the Investigator to review all documentation (e.g. hospital notes, laboratory and diagnostic reports) related to the event. The Investigator will then record all relevant information in the CRF and on the SAE form (if the AE meets the criteria of serious).

Information to be collected includes dose, type of event, onset date, Investigator assessment of severity and causality, date of resolution as well as treatment required, investigations needed and outcome.

10.4 ASSESSMENT OF AEs AND SAEs

Seriousness, causality, severity and expectedness will be assessed by the Principal Investigator or another suitably qualified physician in the research team. For randomised double blind studies, AEs will be assessed as though the participant is taking active IMP. Cases that are considered serious, possibly, probably or definitely related to IMP and unexpected (i.e. SUSARs) will be unblinded.

The Investigator is responsible for assessing each AE. This may be delegated to other suitably qualified physicians in the research team who are trained in recording AEs and recording and reporting SAEs.

The Chief Investigator (CI) may not downgrade an event that has been assessed by an Investigator as an SAE or SUSAR, but can upgrade an AE to an SAE, SAR or SUSAR if appropriate.

10.4.1 Assessment of Seriousness

The Investigator will make an assessment of seriousness as defined in Section 10.1.

10.4.2 Assessment of Causality

The Investigator will make an assessment of whether the AE/SAE is likely to be related to the IMP according to the definitions below.

- Unrelated: where an event is not considered to be related to the IMP.

- Possibly Related: The nature of the event, the underlying medical condition, concomitant medication or temporal relationship make it possible that the AE has a causal relationship to the study drug. The assessment of causality will be made against the reference safety information found in the Summary of Product Characteristics.

Where non Investigational Medicinal Products (NIMPs) e.g. rescue/escape drugs are given: if the AE is considered to be related to an interaction between the IMP and the NIMP, or where the AE might be linked to either the IMP or the NIMP but cannot be clearly attributed to either one of these, the event will be considered as an AR. Alternative causes such as natural history of the underlying disease, other risk factors and the temporal relationship of the event to the treatment should be considered and investigated. The blind should not be broken for the purpose of making this assessment.

10.4.3 Assessment of Expectedness

If an event is judged to be an AR, the evaluation of expectedness will be made based on knowledge of the reaction and the relevant product information documented in the SmPC/IB.

The event may be classed as either:

Expected: the AR is consistent with the toxicity of the IMP listed in the SmPC/IB.

Unexpected: the AR is not consistent with the toxicity in the SmPC/IB.

10.4.4 Assessment of Severity

The Investigator will make an assessment of severity for each AE/SAE and record this on the CRF or SAE form according to one of the following categories:

Mild: an event that is easily tolerated by the participant, causing minimal discomfort and not interfering with every day activities.

Moderate: an event that is sufficiently discomforting to interfere with normal everyday activities.

Severe: an event that prevents normal everyday activities.

Note: the term ‘severe’, used to describe the intensity, should not be confused with ‘serious’ which is a regulatory definition based on participant/event outcome or action criteria. For example, a headache may be severe but not serious, while a minor stroke is serious but may not be severe.

10.5 REPORTING OF SAEs/SARs/SUSARs

Once the Investigator becomes aware that an SAE has occurred in a study participant, the information will be reported to the ACCORD Research Governance & QA Office **immediately or within 24 hours**. If the Investigator does not have all information regarding an SAE, they should not wait for this additional information before notifying ACCORD. The SAE report form can be updated when the additional information is received.

The SAE report will provide an assessment of causality and expectedness at the time of the initial report to ACCORD according to Sections 10.4.2, Assessment of Causality and 10.4.3, Assessment of Expectedness.

The SAE form will be transmitted by fax to ACCORD on **+44 (0)131 242 9447** or may be transmitted by hand to the office or submitted via email to Safety.Accord@ed.ac.uk. Only forms in a pdf format will be accepted by ACCORD via email.

Where missing information has not been sent to ACCORD after an initial report, ACCORD will contact the investigator and request the missing information.

All reports faxed to ACCORD and any follow up information will be retained by the Investigator in the Investigator Site File (ISF).

10.6 REGULATORY REPORTING REQUIREMENTS

The ACCORD Research Governance & QA Office is responsible for pharmacovigilance reporting on behalf of the co-sponsors (Edinburgh University and NHS Lothian).

The ACCORD Research Governance & QA Office has a legal responsibility to notify the regulatory competent authority and relevant ethics committee (Research Ethics Committee (REC) that approved the trial). Fatal or life threatening SUSARs will be reported no later than 7 calendar days and all other SUSARs will be reported no later than 15 calendar days after ACCORD is first aware of the reaction.

ACCORD will inform Investigators at participating sites of all SUSARs and any other arising safety information.

An Annual Safety Report/Development Safety Update Report will be submitted, by ACCORD, to the regulatory authorities and RECs listing all SARs and SUSARs.

10.7 FOLLOW UP PROCEDURES

After initially recording an AE or recording and reporting an SAE, the Investigator should make every effort to follow each event until resolution or death of the participant. Follow up information on an SAE will be reported to the ACCORD office.

11 PREGNANCY

Pregnancy is not considered an AE or SAE; however, the Investigator will collect pregnancy information for any female participants or female partners of male participants who become pregnant while participating in the study. The Investigator will record the information on a Pregnancy Notification Form and submit this to the ACCORD office within 14 days of being made aware of the pregnancy.

All pregnant female participants and partners of male participants will be followed up until following the outcome of the pregnancy.

12 TRIAL MANAGEMENT AND OVERSIGHT ARRANGEMENTS

12.1 TRIAL MANAGEMENT GROUP

The trial will be coordinated by a Project Management Group, consisting of the grant holders (Chief Investigator and Principal Investigator in Edinburgh), a Trial Manager and a coordinating nurse.

The Principal Investigator will oversee the study and will be accountable to the Chief Investigator. The Principal Investigator will be responsible for checking the CRFs for completeness, plausibility and consistency. Any queries will be resolved by the Investigator or delegated member of the trial team.

A Delegation Log will be prepared, detailing the responsibilities of each member of staff working on the trial.

12.2 TRIAL STEERING COMMITTEE

A Trial Steering Committee will be established to oversee the conduct and progress of the trial. Contact details of the TSC are detailed in a separate TSC charter. The review of SAEs will be included in the TSC agenda to ensure that appropriate action is taken if any safety issues arise.

12.3 Data Monitoring Committee

Given the small size of this trial in drugs with a marketing authorisation, it is not been deemed necessary to have a data monitoring committee.

12.4 INSPECTION OF RECORDS

Investigators and institutions involved in the study will permit trial related monitoring and audits on behalf of the sponsor, REC review, and regulatory inspection(s). In the event of an audit or monitoring, the Investigator agrees to allow the representatives of the sponsor direct access to all study records and source documentation. In the event of regulatory inspection, the Investigator agrees to allow inspectors direct access to all study records and source documentation.

12.5 RISK ASSESSMENT

An independent risk assessment will be performed and an ACCORD Clinical Trials Monitor will determine if monitoring is required and if so, at what level. An independent risk assessment will also be carried out by the ACCORD Quality Assurance Group to determine if an audit should be performed before/during/after the study and if so, at what locations and at what frequency.

12.6 BENEFIT/RISK BALANCE

12.6.1 Benefits

Patients may benefit from the treatment intervention that could delay the progression of calcific aortic stenosis. This is particularly relevant for patients who would make poor surgical candidates. Patients may also benefit from additional procedures and investigations that they will undergo as part of the study. This will include closer medical supervision and non-invasive imaging investigations that may identify important incidental findings.

12.6.2 Risks

The main risks will include those from exposure to the study drug, ionising radiation and contrast agent.

In the FREEDOM trial, whilst the overall incidence of cellulitis was similar (1.2% versus 0.9%), there was an excess of cellulitis leading to a serious adverse event (0.3% versus <0.1%, P=0.002). The significance of this remains unclear. However, this will be a particular focus of the safety reporting of the trial. As with bisphosphonates, osteonecrosis of the jaw is rarely seen with denosumab, indeed there were no reported cases of this condition in the FREEDOM trial. Risk factors for this rare but serious adverse event are poor oral hygiene, dental extractions, and co-morbid disorders, especially patients with cancer receiving high-dose denosumab. We will refer patients to a dentist before study enrolment if poor dental hygiene is identified. Patients will not be recruited within 6 months of a dental extraction (if the patient cannot confirm this, with their permission we will contact their dentist). We will exclude patients with major or untreated cancers. We will specifically use the 60 mg dose of denosumab administered to patients with osteoporosis where osteonecrosis of the jaw was not reported. We will also ensure that patients are not hypocalcaemic or vitamin D deficient prior to randomisation. This will be addressed appropriately where necessary by oral supplementation. Calcium levels will be monitored throughout the trial.

There are some potential hazards of the non-invasive investigations that we will perform as part of the trial. The main issues relate to exposure to ionising radiation and contrast agent administration. We have a well-developed protocol for cardiac positron emission and computed tomography imaging that minimizes radiation exposure and has clear procedures for managing adverse contrast reactions.

The total research protocol dose has been estimated at 35mSv. The estimated associated risk of developing fatal cancer is proportional to dose. Using a risk of 5% per Sv in a healthy population in this age group the estimated associated risk of developing fatal cancer as a result of this exposure is in the region 1 in 550. This risk can be classified as moderate. It is likely that in a population of any patients in the age group 50+ the cancer risk is lower than 5% per Sv.

For comparison the average annual background radiation dose arising from natural sources of ionising radiation in the environment in the UK is 2.2mSv. The TRPD of 35mSv incurred in this study is approximately 16 times annual background radiation from natural sources. For those patients entering into the reproducibility studies only. They will be exposed to less radiation, estimated 26mSV which is approximately 12 times that of annual background radiation.

The risks of exposure to the contrast medium include allergic reactions and impairment of kidney function. Amongst patients with moderate-to-severe chronic kidney disease, there is a 2-4% risk of kidney impairment after computed tomography angiography [Barrett *et al*, 2006]. The risk of contrast exposure in this study will be minimised by exclusion of high-risk patients who have significant kidney disease (estimated glomerular filtration rate <30 mL/min/1.73m^2).

12.7 STUDY MONITORING AND AUDIT

An ACCORD Clinical Trials Monitor or an appointed monitor will visit the Investigator site prior to the start of the study and during the course of the study if required, in accordance with the monitoring plan if required. Risk assessment will determine if audit, by the ACCORD QA group, is required. Details will be captured in an audit plan. Audit of Investigator sites, study management activities and study collaborative units, facilities and 3rd parties may be performed.

13 GOOD CLINICAL PRACTICE

13.1 ETHICAL CONDUCT

The study will be conducted in accordance with the principles of the International Conference on Harmonisation Tripartite Guideline for Good Clinical Practice (ICH GCP).

A favorable ethical opinion will be obtained from the appropriate REC and local R&D approval will be obtained prior to commencement of the study.

13.2 REGULATORY COMPLIANCE

The study will not commence until a Clinical Trial Authorisation (CTA) is obtained from the appropriate Regulatory Authority. The protocol and study conduct will comply with the Medicines for Human Use (Clinical Trials) Regulations 2004, as amended.

13.3 INVESTIGATOR RESPONSIBILITIES

The Investigator is responsible for the overall conduct of the study at the site and compliance with the protocol and any protocol amendments. In accordance with the principles of ICH GCP, the following areas listed in this section are also the responsibility of the Investigator. Responsibilities may be delegated to an appropriate member of study site staff.

13.3.1 Informed Consent

The Investigator is responsible for ensuring informed consent is obtained before any protocol specific procedures are carried out. The decision of a participant to participate in clinical research is voluntary and should be based on a clear understanding of what is involved.

Participants must receive adequate oral and written information – appropriate Participant Information and Informed Consent Forms will be provided. The oral explanation to the participant will be performed by the Investigator or qualified delegated person, and must cover all the elements specified in the Participant Information Sheet and Consent Form.

The participant must be given every opportunity to clarify any points they do not understand and, if necessary, ask for more information. The participant must be given sufficient time to consider the information provided. It should be emphasised that the participant may withdraw their consent to participate at any time without loss of benefits to which they otherwise would be entitled.

The participant will be informed and agree to their medical records being inspected by regulatory authorities and representatives of the sponsor(s) but understand that their name will not be disclosed outside the hospital.

The Investigator or delegated member of the trial team and the participant will sign and date the Informed Consent Form(s) to confirm that consent has been obtained. The participant will receive a copy of this document and a copy filed in the Investigator Site File (ISF) and participant's medical notes.

13.3.2 Study Site Staff

The Investigator must be familiar with the IMP, protocol and the study requirements. It is the Investigator's responsibility to ensure that all staff assisting with the study are adequately informed about the IMP, protocol and their trial related duties.

13.3.3 Data Recording

The Principal Investigator is responsible for the quality of the data recorded in the CRF at each Investigator Site. The source data plan identifies which source data correspond to CRF data and states which data are recorded directly into the CRF.

13.3.4 Investigator Documentation

Prior to beginning the study, each Investigator will be asked to provide particular essential documents to the ACCORD Research Governance & QA Office, including but not limited to:

- An original signed Investigator's Declaration (as part of the Clinical Trial Agreement documents);
- Curriculum vitae (CV) signed and dated by the Investigator indicating that it is accurate and current.

The ACCORD Research Governance & QA Office will ensure all other documents required by ICH GCP are retained in a Trial Master File (TMF), where required, and that appropriate documentation is available in local ISFs.

13.3.5 GCP Training

All study staff must hold evidence of appropriate GCP training.

13.3.6 Confidentiality

All laboratory specimens, evaluation forms, reports, and other records must be identified in a manner designed to maintain participant confidentiality. All records must be kept in a secure storage area with limited access. Clinical information will not be released without the written permission of the participant. The Investigator and study site staff involved with this study may not disclose or use for any purpose other than performance of the study, any data, record, or other unpublished, confidential information disclosed to those individuals for the purpose of the study. Prior written agreement from the sponsor or its designee must be obtained for the disclosure of any said confidential information to other parties.

13.3.7 Data Protection

All Investigators and study site staff involved with this study must comply with the requirements of the Data Protection Act 1998 with regard to the collection, storage, processing and disclosure of personal information and will uphold the Act's core principles. Access to collated participant data will be restricted to those clinicians treating the participants, representatives of the sponsor(s) and representatives of regulatory authorities.

Computers used to collate the data will have limited access measures via user names and passwords.

Published results will not contain any personal data that could allow identification of individual participants.

14 STUDY CONDUCT RESPONSIBILITIES

14.1 PROTOCOL AMENDMENTS

Any changes in research activity, except those necessary to remove an apparent, immediate hazard to the participant in the case of an urgent safety measure, must be reviewed and approved by the Chief Investigator.

Amendments to the protocol must be submitted in writing to the appropriate REC, Regulatory Authority and local R&D for approval prior to participants being enrolled into an amended protocol.

14.2 PROTOCOL VIOLATIONS AND DEVIATIONS

Prospective protocol deviations, i.e. protocol waivers, will not be approved by the sponsors and therefore will not be implemented, except where necessary to eliminate an immediate hazard to study participants. If this necessitates a subsequent protocol amendment, this should be submitted to the REC, Regulatory Authority and local R&D for review and approval if appropriate.

Protocol deviations will be recorded in a protocol deviation log and logs will be submitted to the sponsors every 3 months. Each protocol violation will be reported to the sponsor within 3 days of becoming aware of the violation.

14.3 SERIOUS BREACH REQUIREMENTS

A serious breach is a breach that is likely to effect to a significant degree:

(a) the safety or physical or mental integrity of the participants of the trial; or

(b) the scientific value of the trial.

If a potential serious breach is identified by the Chief investigator, Principal Investigator or delegates, the co-sponsors (accord.seriousbreach@ed.ac.uk) must be notified within 24 hours. It is the responsibility of the co-sponsors to assess the impact of the breach on the scientific value of the trial, to determine whether the incident constitutes a serious breach and report to regulatory authorities and research ethics committees as necessary.

14.4 STUDY RECORD RETENTION

All study documentation will be kept for a minimum of 5 years from the protocol defined end of study point. Patient identifiable information will be kept beyond 5 years to facilitate longer term follow up if necessary (this will be made explicit on consent forms and patient information sheets). When the minimum retention period has elapsed, study documentation will not be destroyed without permission from the sponsor.

14.5 END OF STUDY

It is anticipated that the study will last 3 years: one year for recruitment and two years of follow-up. The end of study is defined as the last participant's last visit.

The Investigators, trial management group and/or the co-sponsor(s) have the right at any time to terminate the study for clinical or administrative reasons.

The end of the study will be reported to the REC and Regulatory Authority within 90 days, or 15 days if the study is terminated prematurely. The Investigators will inform participants of the premature study closure and ensure that the appropriate follow up is arranged for all participants involved.

A summary report of the study will be provided to the REC and Regulatory Authority within 1 year of the end of the study.

14.6 CONTINUATION OF DRUG FOLLOWING THE END OF STUDY

The study medication will not be continued at the trial conclusion as they are not currently licensed for use in patients with calcific aortic stenosis.

14.7 INSURANCE AND INDEMNITY

The co-sponsors are responsible for ensuring proper provision has been made for insurance or indemnity to cover their liability and the liability of the Chief Investigator and staff.

The following arrangements are in place to fulfil the co-sponsors' responsibilities:

- The Protocol has been designed by the Chief Investigator and researchers employed by the University and collaborators. The University has insurance in place (which includes no-fault compensation) for negligent harm caused by poor protocol design by the Chief Investigator and researchers employed by the University.
- Sites participating in the study will be liable for clinical negligence and other negligent harm to individuals taking part in the study and covered by the duty of care owed to them by the sites concerned. The co-sponsors require individual sites participating in the study to arrange for their own insurance or indemnity in respect of these liabilities.
- Sites which are part of the United Kingdom's Nation Health Service will have the benefit of NHS Indemnity.
- Sites out with the United Kingdom will be responsible for arranging their own indemnity or insurance for their participation in the study, as well as for compliance with local law applicable to their participation in the study.
- The manufacturer supplying IMP has accepted limited liability related to the manufacturing and original packaging of the study drug and to the losses, damages, claims or liabilities incurred by study participants based on known or unknown Adverse Events which arise out of the manufacturing and original packaging of the study drug, but not where there is any modification to the study drug (including without limitation re-packaging and blinding).

15 REPORTING, PUBLICATIONS AND NOTIFICATION OF RESULTS

15.1 AUTHORSHIP POLICY

Ownership of the data arising from this study resides with the study team. On completion of the study, the study data will be analysed and tabulated, and a clinical study report will be prepared in accordance with ICH guidelines.

15.2 PUBLICATION

The clinical study report will be used for publication and presentation at scientific meetings. Investigators have the right to publish orally or in writing the results of the study.

Summaries of results will also be made available to Investigators for dissemination within their clinics (where appropriate and according to their discretion).

15.3 PEER REVIEW

The study protocol has undergone independent review by an Edinburgh Clinical Trials Unit statistician, and has undergone by the British Heart Foundation. The protocol has been amended in accordance with their recommendations.

APPENDIX 1: Trial Management Group

Chief Investigator:	Professor David Newby
Supervising Principle Investigator:	Dr Marc Dweck
Principle Investigator:	Dr Rong Bing

Appendix 2: SALTIRE II Study Flow Chart

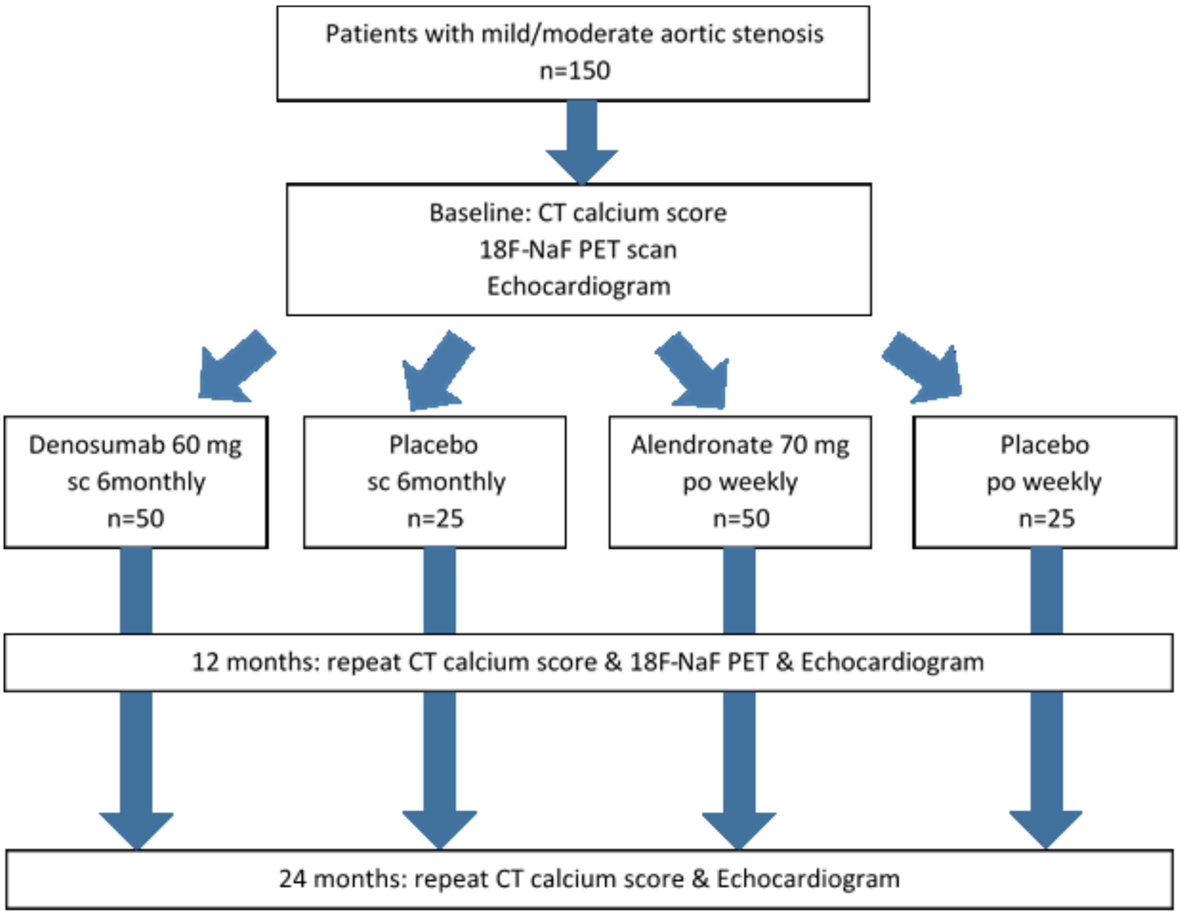

APPENDIX 3: References

1. Kaden JJ, Bickelhaupt S, Grobholz R, Haase KK, Sarikoc A, Kilic R, et al. Receptor activator of nuclear factor kappaB ligand and osteoprotegerin regulate aortic valve calcification. Journal of molecular and cellular cardiology. 2004;36(1):57-66.
2. Rajamannan NM, Subramaniam M, Rickard D, Stock SR, Donovan J, Springett M, et al. Human Aortic Valve Calcification Is Associated With an Osteoblast Phenotype. Circulation. 2003;107(17):2181-4.
3. Cowell SJ, Newby DE, Prescott RJ, Bloomfield P, Reid J, Northridge DB, et al. A randomized trial of intensive lipid-lowering therapy in calcific aortic stenosis. The New England journal of medicine. 2005;352(23):2389-97.
4. Cowell SJ, Newby DE, Burton J, White A, Northridge DB, Boon NA, et al. Aortic valve calcification on computed tomography predicts the severity of aortic stenosis. Clinical radiology. 2003;58(9):712-6.
5. Rossebo AB, Pedersen TR, Boman K, Brudi P, Chambers JB, Egstrup K, et al. Intensive lipid lowering with simvastatin and ezetimibe in aortic stenosis. The New England journal of medicine. 2008;359(13):1343-56.
6. Chan KL, Teo K, Dumesnil JG, Ni A, Tam J, Investigators A. Effect of Lipid lowering with rosuvastatin on progression of aortic stenosis: results of the aortic stenosis progression observation: measuring effects of rosuvastatin (ASTRONOMER) trial. Circulation. 2010;121(2):306-14.
7. Rudd JHF, Narula J, Strauss HW, Virmani R, Machac J, Klimas M, et al. Imaging Atherosclerotic Plaque Inflammation by Fluorodeoxyglucose With Positron Emission Tomography: Ready for Prime Time? Journal of the American College of Cardiology. 2010;55(23):2527-35.
8. Dweck MR, Jenkins WSA, Vesey AT, Pringle MAH, Chin CWL, Malley TS, et al. 18F-NaF Uptake Is a Marker of Active Calcification and Disease Progression in Patients with Aortic Stenosis. Circulation: Cardiovascular Imaging. 2014.
9. Dweck MR, Jenkins WS, Vesey AT, Pringle MA, Chin CW, Malley TS, et al. 18F-NaF Uptake Is a Marker of Active Calcification and Disease Progression in Patients with Aortic Stenosis. Circulation Cardiovascular imaging. 2014.
10. Dweck MR, Khaw HJ, Sng GKZ, Luo ELC, Baird A, Williams MC, et al. Aortic stenosis, atherosclerosis, and skeletal bone: is there a common link with calcification and inflammation? European heart journal. 2013;34(21):1567-74.
11. Dweck MR, Jones C, Joshi NV, Fletcher AM, Richardson H, White A, et al. Assessment of valvular calcification and inflammation by positron emission tomography in patients with aortic stenosis. Circulation. 2012;125(1):76-86.
12. Aksoy Y, Yagmur C, Tekin GO, Yagmur J, Topal E, Kekilli E, et al. Aortic valve calcification: association with bone mineral density and cardiovascular risk factors. Coronary Artery Disease. 2005;16(6):379-83.
13. Demer LL, Tintut Y. Vascular Calcification: Pathobiology of a Multifaceted Disease. Circulation. 2008;117(22):2938-48.
14. Persy V, D'Haese P. Vascular calcification and bone disease: the calcification paradox. Trends in Molecular Medicine. 2009;15(9):405-16.
15. Price PA, Faus SA, Williamson MK. Bisphosphonates Alendronate and Ibandronate Inhibit Artery Calcification at Doses Comparable to Those That Inhibit Bone Resorption. Arteriosclerosis, Thrombosis, and Vascular Biology. 2001;21(5):817-24.
16. Rosenblum IY, Flora L, Eisenstein R. The effect of disodium ethane-1-hydroxy-1,1-diphosphonate (EHDP) on a rabbit model of athero-arteriosclerosis. Atherosclerosis. 1975;22(3):411-24.
17. Kramsch DM, Chan CT. The effect of agents interfering with soft tissue calcification and cell proliferation on calcific fibrous-fatty plaques in rabbits. Circulation research. 1978;42(4):562-71.
18. Price PA, Faus SA, Williamson MK. Bisphosphonates alendronate and ibandronate inhibit artery calcification at doses comparable to those that inhibit bone resorption. Arteriosclerosis, thrombosis, and vascular biology. 2001;21(5):817-24.

19. Sansoni P, Passeri G, Fagnoni F, Mohagheghpour N, Snelli G, Brianti V, et al. Inhibition of antigen-presenting cell function by alendronate in vitro. Journal of bone and mineral research : the official journal of the American Society for Bone and Mineral Research. 1995;10(11):1719-25.
20. Giraudo E, Inoue M, Hanahan D. An amino-bisphosphonate targets MMP-9-expressing macrophages and angiogenesis to impair cervical carcinogenesis. The Journal of clinical investigation. 2004;114(5):623-33.
21. Lai TJ, Hsu SF, Li TM, Hsu HC, Lin JG, Hsu CJ, et al. Alendronate inhibits cell invasion and MMP-2 secretion in human chondrosarcoma cell line. Acta pharmacologica Sinica. 2007;28(8):1231-5.
22. Elmariah S, Delaney JA, O'Brien KD, Budoff MJ, Vogel-Claussen J, Fuster V, et al. Bisphosphonate Use and Prevalence of Valvular and Vascular Calcification in Women MESA (The Multi-Ethnic Study of Atherosclerosis). Journal of the American College of Cardiology. 2010;56(21):1752-9.
23. Rapoport HS, Connolly JM, Fulmer J, Dai N, Murti BH, Gorman RC, et al. Mechanisms of the in vivo inhibition of calcification of bioprosthetic porcine aortic valve cusps and aortic wall with triglycidylamine/mercapto bisphosphonate. Biomaterials. 2007;28(4):690-9.
24. Sattler AM, Schoppet M, Schaefer JR, Hofbauer LC. Novel aspects on RANK ligand and osteoprotegerin in osteoporosis and vascular disease. Calcified tissue international. 2004;74(1):103-6.
25. Bucay N, Sarosi I, Dunstan CR, Morony S, Tarpley J, Capparelli C, et al. osteoprotegerin-deficient mice develop early onset osteoporosis and arterial calcification. Genes & development. 1998;12(9):1260-8.
26. Morony S, Tintut Y, Zhang Z, Cattley RC, Van G, Dwyer D, et al. Osteoprotegerin inhibits vascular calcification without affecting atherosclerosis in ldlr(-/-) mice. Circulation. 2008;117(3):411-20.
27. Cummings SR, San Martin J, McClung MR, Siris ES, Eastell R, Reid IR, et al. Denosumab for prevention of fractures in postmenopausal women with osteoporosis. The New England journal of medicine. 2009;361(8):756-65.
28. Helas S, Goettsch C, Schoppet M, Zeitz U, Hempel U, Morawietz H, et al. Inhibition of receptor activator of NF-kappaB ligand by denosumab attenuates vascular calcium deposition in mice. The American journal of pathology. 2009;175(2):473-8.
29. Dweck MR, Khaw HJ, Sng GK, Luo EL, Baird A, Williams MC, et al. Aortic stenosis, atherosclerosis, and skeletal bone: is there a common link with calcification and inflammation? European heart journal. 2013;34(21):1567-74.
30. Dweck MR, Boon NA, Newby DE. Calcific aortic stenosis: a disease of the valve and the myocardium. Journal of the American College of Cardiology. 2012;60(19):1854-63.

Protocol Version	Date	Details of Amendment
V1.1	6th May 2014	N/A
V1.7	29th September 2014	• Addition of a secondary endpoint for the reproducibility sub study.<br>• Addition of clinical history and physical examination in the reproducibility sub study to ensure patients entering into the sub study reflected the patient population as a whole.<br>• Addition of a 2-week phone call to the alendronate arm of the study.<br>• Correction of the minimum age to >50.<br>• Reduction in the time between identify and consenting a patient from 7 days to 2 days.<br>• Clarification of the blinding methods.<br>• Update to the alendronate manufacturing details to include a new manufacturer.<br>• Guidance included for patients who commenced on treatment for osteoporosis during the course of the trial.<br>• Addition of the sample size calculation methods and analysis methods for the reproducibility sub study.
V2.0	3rd March 2015	• Addition of a 2-week phone call to the denosumab arm of the study, in accordance with new guidelines regarding monitoring for hypocalcaemia.<br>• Guidance included for monitoring of hypocalcaemia including re-measuring a participant's vitamin D levels if they developed hypocalcaemia during the study.<br>• Combination of screening and baseline visits to enable these to be performed on the same day in order to minimise trial burden on the participant.<br>• Addition of the beta-blockers, which were given during the 18-NaF PET/CT imaging, in the NIMP section.<br>• Update to the biochemical parameters measured at the screening visit.<br>• Addition of an optional genetic blood sample for future research into the genetics of aortic stenosis.

		• Clarification included to confirm that where it was possible for acceptable reproducibility in a smaller number of patients in the reproducibility sub-study then recruitment to the sub study would stopped.<br>• Clarification about pre-existing medical conditions and symptoms of the participant's underlying disease included in the Adverse Events Section.
V2.1	10th June 2015	Addition of an additional minimisation criterion in the randomisation algorithm for the main study.
V3.0	10th January 2016	Movement of the 2nd 18F-Fluoride PET scan from the 6-month visit to the 12-month visit.
V4.0	14th September 2016	Removal of the summary of product characteristics hyperlinks.
V5.0	29th November 2016	Guidance included for participants who needed to undergo aortic valve replacement surgery within 24 months of randomisation.
V6.0	10th August 2017	Change to Principal Investigator.
V7.0	24th October 2017	Clarification added for measuring/checking calcium levels prior to administering Denosumab.
V8.0	30th August 2018	Change to the manufacturing authorisation number for the Alendronic tablets due to the original marketing authorisation number being discontinued.
V9.0	2nd July 2019	Change to Principal Investigator.
V10.0	19th August 2019	Clarification added to the emergency unblinding section of the protocol.<br>Clarification added to the follow-up procedures section of the protocol.<br>Following review of the above updates, the MHRA requested further clarification and for an updated version of the protocol be re-submitted (V11 29th October 2019). As a result, V10.0 of the protocol was not implemented.
V11.0	29th October 2019	N/A

Bisphosphonates and RANKL inhibition in Aortic Stenosis [The SALTIRE II Trial]

Statistical Analysis Plan

**Version No**	3.2
**Date**	17 April 2020
**Author(s)**	C.Graham
**CI Name**	Professor David E Newby
**CI Contact Details**	Centre of Cardiovascular Science<br>Chancellor's Building<br>51 Little France Crescent<br>Edinburgh EH16 4SB<br>Tel: 0131 242 6515<br>Email: d.e.newby@ed.ac.uk
**EudraCR-Number**	2014-001112-19
**REC Number**	14/SS/0064
**Protocol based on**	Version 11, 29 October2019

Signatures	
**Trial Statistician: C.Graham**	**Date: 17Apr2020**
**Chief Investigator:**<br>**David E. Newby**	**Date: 17th April 2020**

**Document Control**		
**Version No**	**Date**	**Summary of Revisions**
1.0	21May2015	Initial Creation
2.0	16Nov2015	
3	22Oct2019	
3.1	06Apr2020	Move to new template, updated based on final version of trial protocol.

3.2	17Apr2020	Updates to reflect CI/PI comments.

Table of Contents

List of Abbreviations 3
1. Introduction 4
2. Statistical Methods section from the protocol 4
3. Overall Statistical Principles 5
3.1 Analysis populations 5
3.2 Treatment allocation 5
3.3 Compliance 5
3.4 Analysis considerations 6
3.5 Interim analysis 6
3.6 Timing of final analysis 7
3.7 Missing data 7
4. List of Analyses 7
4.1 Reproducibility sub-study 7
4.2 Main study 8
4.3 Comparison of placebo groups 10
5. Validation and QC 10
6. References 10

List of Abbreviations

Abbreviation	Full name
18F-NaF	18-F fluoride
AE	Adverse event
CT	Computed tomography
FDG	Fluorodeoxyglucose
MDS	Most diseased segment
PET	Positron emission tomography
SAE	Serious adverse event
SF-36	Short form 36
TBR	Tissue background ration
TSC	Trial steering committee
V_{max}	Velocity maximum

1. Introduction

This will be a single centre double blind parallel group randomised placebo-controlled trial of alendronate and denosumab in patients with calcific aortic stenosis. A reproducibility sub-study will be conducted on 20 patients who meet the eligibility criteria (not included in the main trial). The main trial aims to recruit 150 participants allocated to treatment arm using minimisation with the following factors: age [<73 and ≥73 years], sex, presence or absence of a bicuspid valve and baseline aortic valve calcium scores (≤1607, >1607 Agatston Units). The minimisation will allocate participants to the group which minimises the treatment imbalance with a built in random component to ensure the minimisation allocation is random in a 2:1:2:1 ratio to alendronate: alendronate placebo: denosumab: denosumab placebo.

2. Statistical Methods section from the protocol

Description of Analysis Sets
Main Study
For the purpose of analysis, we will retain participants in the treatment groups to which they were originally assigned irrespective of the treatment actually received with the exception of adverse event data which will be presented by treatment received. For patients who undergo aortic valve replacement surgery within 24 months of randomisation, we will bring forward and perform their 12-month or 24-month visit prior to discontinuation of study medication and the conduct of surgery. These patients will be retained within the analysis. All patients withdrawn from the study that have unresolved (S)AEs will be monitored until resolution of the event or until no longer medically indicated.
Methods of Statistical Analysis: Main Study
Descriptive analysis of participant characteristics will be presented split by treatment allocation. These analyses will be performed comparing alendronate versus placebo and separately for denosumab versus placebo.
Baseline to two-year change will be analysed using two sample t-tests or non-parametric equivalent as appropriate: aortic valve score, aortic valve NaF uptake, aortic jet velocity, thoracic calcium score, coronary calcium score, bone mineral density and SF-36. It is known that calcium scores tend to follow a skewed distribution and often a log-transformation is sufficient to result in normally distributed data, before using a non-parametric method appropriate transformations will be explored and utilised if appropriate.

Reproducibility Substudy
With respect to 18F-NaF, the PET tracer uptake is quoted as a tissue to background ratio (TBR). An expected treatment effect of 40% should result in a (TBR) change of 0.5. This is based on the difference in the activity between patients with moderate disease compared to the controls from our previous work: moderate (Max TBR) 2.89 SD 2.31-3.24, control (Max TBR) 1.56 SD 1.41-1.64. Results in a similar reproducibility study for FDG showed the width of the 95% limits of agreement of ± 0.1-0.2 with respect to the max TBR in 11 patients. These data will be presented and analysed using a Bland Altman

Analysis. If it is possible to demonstrate reproducibility by the 95% limits of agreement within ± 0.2 TBR within the first 15 patients we will proceed to the main study. If the 95% limits of agreement are ± 0.4 TBR then we will proceed to recruit 20 patients. If we are unable to demonstrate reproducibility, i.e. 95% limits of agreement greater than ± 0.4 TBR in 20 patients then we will review the protocol of the main study before proceeding. These decisions to proceed with the main trial will be made by the Trial Steering Committee.

3. Overall Statistical Principles

Unless otherwise stated, for all analysis, the level of significance will be taken as two-sided p=0.05.

3.1 Analysis populations

Reproducibility sub-study: first 15 participants recruited for the purpose of the reproducibility sub-study. This group may be extended to 20 participants following TSC review. These participants will not be included in the analysis of the main trial.

Main study intention to treat: all participants who are considered to have been recruited into the trial, i.e. those who have attended for their randomisation visit, in the groups to which they were originally assigned irrespective of treatment received.

Main study per protocol 50% compliant: all participants who are considered to have been recruited into the trial and are considered to be compliant with trial procedures as defined in the compliance section below to a level of at least 50%.

Main study per protocol 70% compliant: all participants who are considered to have been recruited into the trial and are considered to be compliant with trial procedures as defined in the compliance section below to a level of at least 70%.

3.2 Treatment allocation

For all analysis, unless otherwise specified, participants shall be analysed according to the treatment group they were originally assigned irrespective of treatment received. The number of participants where the treatment first received is not the same as the treatment allocated shall be recorded and reason for this change documented.

3.3 Compliance

From the point of view of the compliance analysis, we will consider all participants who have completed a minimum of 50% (i.e. greater than or equal to 50% compliant). For the different arms of the study how this is determined will be:

- Injection arms: at least two or more injections over the study duration (maximum of 4 if participant remains in the study for the full two-year duration).
- Tablet arms: participants are issued with 32 tablets at each six-monthly visit which covers the planned one tablet per week with 6 extra tablets to allow for a delay in attending the next

study visit. Over the entire study duration, each person would receive a total of 128 tablets and to be considered compliant must have taken at least 52. Participants were asked to return any unused tablets at the next study visit and, where this occurred, the number of pills returned were counted. However, participants often did not return unused pills for counting at the next visit, in some cases they may have brought them to subsequent visits although it is not always clear where this is the case.

- Number of pill taken will be determined by [32 (the number of pills issued at each visit) * number of visits] – number of pills returned

3.4 Analysis considerations

3.4.1 Placebo group

There are two placebo groups in this study: two groups of n=25 with placebo tablets or placebo injections. For the purpose of the sample size calculation, it has been assumed that these groups can be combined to form a single placebo group. The analysis as described assumes that these two groups are treated as a single placebo group.

Given the size of the placebo groups, we are not powered to detect a difference between them.

3.4.2 Calcium score transformation

If appropriate the calcium score at each time point will be log transformed. Any subsequent analysis where it is described in the remainder of this document that a calcium score will be analysed, whether this a point estimate or a change in value, will be taken to be an analysis of the log transformed point estimate or a change in the log transformed values.

3.4.3 Change from baseline

The change will be expressed as either the baseline to 12-month change (PET variables- aortic valve NaF uptake, thoracic aorta NaF uptake and coronary NaF uptake) or baseline to 24-month change. In some instances, participants have had their 12-month or 24-month visit brought forward so that data is captured prior to the person undergoing aortic valve replacement surgery. In order to account for this, we will determine the baseline to end change in values, calculate the number of days between the baseline and end measurement and express this as a change over one or two years using the following method:

Baseline to one year change: ((12-month value - base value) / (12-month visit date - base date)) * 365

Baseline to two year change: ((end value - base value) / (end date - base date)) * 730

3.5 Interim analysis

The drugs already have marketing authorisation for other indications, the number of participants modest and any treatment and safety signals are extremely unlikely to be identifiable during the conduct of the trial. There shall be no formal Data Monitoring Committee and as such there will be no formal interim analysis of the main phase of this study.

3.6 Timing of final analysis

The reproducibility sub study data shall be analysed for the 15 eligible participants. After review, the TSC will decide whether a further 5 participants are required or to proceed with recruitment for the main study.

The analysis for the main study will only be performed at study completion. It will be performed on the dataset after any 'cleaning' that may be required has been completed and the database locked.

3.7 Missing data

As the study is being overseen by the Clinical Trials Unit, it is expected that missing data will be at a minimum as any missing data will be routinely queried. We will indicate the number of participants with missing information and where necessary we may consider on a case-by-case basis additional methods of dealing with missing key observations which have high levels of missing data.

In an attempt to minimise missing data, where a participant is scheduled to undergo aortic valve replacement surgery prior to the 24-month visit the 24-month (or 12-month visit if not yet reached that time point) will be brought forward and conducted prior to surgery. In this way, it will be possible to use readings obtained at that point correcting for the duration between baseline and follow up.

SF-36: there are multiple questions that make up each domain in the SF-36 and it is not possible to calculate a domain score if one/some of the responses are missing. If there are missing responses, where it is possible to calculate the domain score, this will be done using the method described by in SF-36 scoring manual (Ware *et al*).

- Where at least half the items are completed (or half + 1, in scales with an odd number of items).
- Blank items are replaced with a score that is the average of all completed items in that domain.

4. List of Analyses

4.1 Reproducibility sub-study

1. Number (and percentage) of participants completing each section of the study.

2. Descriptive analysis of the participants will be presented for: participant demographics, vital signs, symptoms, physical examination and relevant medical history. For categorical data, this will consist of number and percentage and where data are continuous, we will present mean, standard deviation, minimum, 25th centile, median, 75th centile and maximum.

3. Laboratory tests, descriptive analysis of blood markers will be presented graphically showing the baseline to two-week change within each individual. As well as descriptive statistics of each markers: mean, standard deviation, minimum, 25th centile, median, 75th centile and maximum.

4. Maximum TBR at baseline and 2-week follow up will be presented graphically using a Bland-Altman plot. This will display the mean difference along with 95% limits of agreement. Descriptive statistics will also be presented for the baseline and 2-week follow up: mean, standard deviation, minimum, 25th centile, median, 75th centile and maximum.

5. Adverse events – the number and percentage of participants who experience AEs will be presented along with a line listing providing a detailed description of each AE.

6. Concomitant medications – the number and percentage of participants who are receiving concomitant medications at each time point. A line listing of medication details will be presented including medication name, dose, start date/end date and any other relevant information present.

4.2 Main study

The following analysis will be an intention to treat analysis.

1. Recruitment – a standard accrual plot showing the cumulative total over time with a planned recruitment line shown to illustrate the projected target. In addition a plot showing the number of participants recruited each quarter.

2. Attendance – for each visit the number and percent of participants will be presented and broken down by treatment. Where a reason for non-attendance is available, this will presented. Descriptive statistics will be presented to illustrate the timing of each visit by treatment.

3. Compliance – number and percent of participants who were allocated to treatment and who received the allocated treatment. Number and percent of participants who are considered to be compliant at each compliance level: minimum of 50% compliant, minimum of 70% compliant. Descriptive statistics for the absolute level of compliance: mean, standard deviation, minimum, 25th centile, median, 75th centile and maximum.

   In addition to this we will present the number (and percent) of participants in the pill arms of this study where we have pills returned at each visit.

4. Baseline demographic variables - participant demographics, vital signs, symptoms, physical examination and relevant medical history will be presented broken down by treatment. For categorical data, this will consist of number and percentage and where data are continuous, we will present: mean, standard deviation, minimum, 25th centile, median, 75th centile and maximum.

   A table will be presented separately from the baseline table showing the distribution of participants according the variables used in the minimisation: age [<73 and ≥73 years], sex, presence or absence of a bicuspid valve and baseline aortic valve calcium scores (≤1607, >1607 Agatston Units). These variables are all expressed in a binary form and will be presented as number of participants and percentage of each treatment allocation.

5. Clinical characteristics (vital signs, echocardiography measurements, 18F-fluoride PET measurements and CT calcium scores) – where data are continuous, descriptive statistics

SAP Template/v2.0/12March2018

(mean, standard deviation, minimum, 25th centile, median, 75th centile and maximum) shall be presented for each time point and the pattern of change over time shall be presented graphically, by treatment. Where data are categorical, the number and percent will be presented at each time point broken down by treatment.

6. Laboratory tests – number and percent of participants with blood samples taken, where reasons provided for non-collection, these will be provided. For each marker, descriptive statistics (mean, standard deviation, minimum, 25th centile, median, 75th centile and maximum) shall be presented for each time point and the pattern of change over time shall be presented graphically, by treatment.

7. Primary outcome analysis – change in aortic valve calcium score will be analysed and presented in a number of ways

    a. Descriptive statistics will be presented at each time point and change over time will be presented graphically by treatment.
    b. Each active treatment will be compared separately to placebo using a two-sample t-test.
    c. To take into account multiple time points, we will perform a mixed effects linear regression of each active treatment with placebo separately. This will be done fitting a mixed model with: treatment, timepoint, and baseline as fixed effects and participant as a random effect. Results presented will be the estimate, standard error, degrees of freedom, t-value and p-value for the differences of least squares means.

8. Secondary outcome analysis – the same analysis as described in part 6 above will be performed for the following variables/time points.
    d. Change in aortic valve 18F fluoride uptake [baseline to 12 months]
    e. Change in aortic jet velocity as defined by aortic valve V_{max} [baseline to 24 months]
    f. Change in thoracic aortic calcium score [baseline to 24 months]
    g. Change in coronary artery calcium score [baseline to 24 months]
    h. Change in thoracic aorta TBR MDS mean [baseline to 12 months]
    i. Change in coronary 18F-fluoride uptake as represented by CMA [baseline to 12 months]
    j. Change in bone mineral density [baseline to 24 months]
    k. Change in quality of life as determined by SF-36 [baseline to 24 months] *mixed regression will not be performed for this variable*. Where the SF-36 will be presented in the form of six domains calculated as per the SF-36 scoring manual (Ware *et al*). In summary, a raw score is calculated for each domain by summing the responses after transposing of required variables and this raw score is then transformed to a score ranging from 0 to 100 using the formula:

        Transformed scale = [(raw score – lowest possible raw score) / possible raw score range] * 100

9. Sensitivity analysis – the primary outcome analysis will be repeated using the 'main study protocol compliant' population, i.e. compliant at a level of at least 50% and separately for compliant to a level of least 70%.

10. Adverse events – the number and percentage of participants in each arm with at least one AE will be presented and if appropriate a comparison of the proportions will be performed.

Descriptive statistics of the number of AEs experienced by each participant (mean, standard deviation, minimum, 25th centile, median, 75th centile and maximum) shall be presented by treatment. A line listing of AEs in each treatment will be provided.

11. Serious adverse events – this information will come from the sponsors pharmacovigilance system and the number and percentage of participants in each arm with an SAE will be presented and if appropriate a comparison of the proportions will be performed. Descriptive statistics of the number of AEs experienced by each participant (mean, standard deviation, minimum, 25th centile, median, 75th centile and maximum) shall be presented by treatment. A line listing of SAEs in each treatment will be provided.

12. Protocol violations & deviations – this information will come from the sponsors violations & deviations reporting system. A line listing will be provided for violations and separately for deviations.

13. Unblinding – the number and percent of participants where unblinding has been performed during the course of the study will be presented by treatment. Reasons for unblinding will be presented with other relevant information in the form of a line listing.

4.3 Comparison of placebo groups

There are two placebo groups in this study (each n=25), for the purpose of the sample size calculation it has been assumed that these groups can be combined to form a single placebo group. The following information will be presented showing the two placebo groups separately.

1. Baseline demographics
2. Clinical characteristics
3. Comparison of the baseline to 24-month change in aortic valve calcium score using a two-sample t-test.

5. Validation and QC

The analysis will be performed after any ‘cleaning’ of the data has concluded and the database locked.

The primary outcome will be independently validated by a second statistician or if that is not possible the primary outcome will be recoded independently of the original programming.

6. References

Ware JE, Kosinski M, Dewney JE. How to Score Version 2 of the SF36 Health Survey. Lincoln, RI. Quality Metric Incorporated, 2000.

SAP Template/v2.0/12March2018
